# Ubiquitin specific peptidase 37 and PCNA interaction promotes osteosarcoma pathogenesis by modulating replication fork progression

**DOI:** 10.1186/s12967-023-04126-2

**Published:** 2023-04-28

**Authors:** Ravi Chauhan, Ashna Gupta, Lakshay Malhotra, Ajaz A. Bhat, Raj K. Pandita, Tariq Masoodi, Gunjan Dagar, Hana Q. Sadida, Sara K. Al-Marzooqi, Atul Batra, Sameer Bakhshi, Mehar Chand Sharma, Pranay Tanwar, Shah Alam Khan, Ethayathulla Abdul Samath, Shahab Uddin, Ammira S. Al-Shabeeb Akil, Mohammad Haris, Muzafar A. Macha, Tej K. Pandita, Mayank Singh

**Affiliations:** 1 Department of Medical Oncology (Lab), Dr. BRAIRCH, All India Institute of Medical Sciences (AIIMS), New Delhi, Delhi 110029 India; 2 Department of Biophysics, All India Institute of Medical Sciences, New Delhi, India; 3 Department of Human Genetics-Precision Medicine in Diabetes, Obesity and Cancer Research Program, Sidra Medicine, Doha, Qatar; 4 Center for Genomics and Precision Medicine, Texas A&M College of Medicine, Houston, TX USA; 5 Laboratory of Cancer Immunology and Genetics, Sidra Medicine, Doha, Qatar; 6 Department of Medical Oncology, All India Institute of Medical Sciences, New Delhi, India; 7 Department of Pathology, All India Institute of Medical Sciences, New Delhi, India; 8 Department of Lab Oncology, Dr. BRAIRCH. All India Institute of Medical Sciences (AIIMS), New Delhi, India; 9 Department of Orthopaedics, Dr. BRAIRCH, All India Institute of Medical Sciences (AIIMS), New Delhi, India; 10 Translational Research Institute, Academic Health System, Hamad Medical Corporation, Doha, Qatar; 11 Center for Advanced Metabolic Imaging in Precision Medicine, Department of Radiology, Perelman School of Medicine, University of Pennsylvania, Philadelphia, USA; 12 Watson-Crick Centre for Molecular Medicine, Islamic University of Science and Technology, Pulwama, India

**Keywords:** Deubiquitinating enzymes, Ubiquitin specific protease 37, Proliferating cell nuclear antigen, Replication stress, Metastasis

## Abstract

**Background:**

Osteosarcoma is a type of bone cancer that predominantly affects young individuals, including children and adolescents. The disease progresses through heterogeneous genetic alterations, and patients often develop pulmonary metastases even after the primary tumors have been surgically removed. Ubiquitin-specific peptidases (USPs) regulate several critical cellular processes, such as cell cycle progression, transcriptional activation, and signal transduction. Various studies have revealed the significance of USP37 in the regulation of replication stress and oncogenesis.

**Methods:**

In this study, the Cancer Genome Atlas (TCGA) database was analyzed to investigate USP37 expression. RNA sequencing was utilized to assess the impact of USP37 overexpression and depletion on gene expression in osteosarcoma cells. Various molecular assays, including colony formation, immunofluorescence, immunoprecipitation, and DNA replication restart, were employed to examine the physical interaction between USP37 and PCNA, as well as its physiological effects in osteosarcoma cells. Additionally, molecular docking studies were conducted to gain insight into the nature of the interaction between USP37 and PCNA. Furthermore, immunohistochemistry was performed on archived tissue blocks from osteosarcoma patients to establish a correlation between USP37 and PCNA expression.

**Results:**

Analysis of the TCGA database revealed that increased expression of USP37 was linked to decreased progression-free survival (PFS) in osteosarcoma patients. Next-generation sequencing analysis of osteosarcoma cells demonstrated that overexpression or knockdown of USP37 led to the expression of different sets of genes. USP37 overexpression provided a survival advantage, while its depletion heightened sensitivity to replication stress in osteosarcoma cells. USP37 was found to physically interact with PCNA, and molecular docking studies indicated that the interaction occurs through unique residues. In response to genotoxic stress, cells that overexpressed USP37 resolved DNA damage foci more quickly than control cells or cells in which USP37 was depleted. The expression of USP37 varied in archived osteosarcoma tissues, with intermediate expression seen in 52% of cases in the cohort examined.

**Conclusion:**

The results of this investigation propose that USP37 plays a vital role in promoting replication stress tolerance in osteosarcoma cells. The interaction between USP37 and PCNA is involved in the regulation of replication stress, and disrupting it could potentially trigger synthetic lethality in osteosarcoma. This study has expanded our knowledge of the mechanism through which USP37 regulates replication stress, and its potential as a therapeutic target in osteosarcoma merits additional exploration.

**Supplementary Information:**

The online version contains supplementary material available at 10.1186/s12967-023-04126-2.

## Introduction

Despite the surgical removal of tumors, oncologists continue to face challenges in treating osteosarcoma, as 80% of patients develop metastasis to the lung. Unfortunately, the systematic development of drugs for osteosarcoma has been stagnant. The standard therapy for osteosarcoma, which involves surgery and chemotherapy, was established in the 1980s and has resulted in long-term survival in more than 60% of patients with localized disease. Cisplatin has been demonstrated to be beneficial as adjuvant therapy in two studies from a decade ago, and continues to serve as the cornerstone of adjuvant chemotherapy [1].

For the past four decades, the three-drug chemotherapy regimen commonly used to treat localized osteosarcoma has produced consistent outcomes for patients. The most frequently used chemotherapy protocol, MAP, involves administering high-dose methotrexate, doxorubicin (adriamycin), and cisplatin both before and after surgery. Clinical trials have explored different combinations of the five chemotherapeutic agents known to be effective in treating this disease, namely methotrexate, doxorubicin, cisplatin, ifosfamide, and etoposide [2–5]. Unfortunately, the results of osteosarcoma treatment have been less than optimal, and numerous studies over the past four decades have highlighted the significant heterogeneity present at the molecular level. Spectral karyotyping has uncovered a vast array of chromosomal and structural abnormalities, including translocations that result in complex derivative chromosomes and the formation of numerous fusion sequences [6, 7]. One hallmark characteristic of osteosarcoma is the upregulation of MYC, which occurs in nearly 41% of cases and is linked to an elevated risk of metastasis and reduced survival rates in patients with this disease [8].

The ubiquitin proteasomal system has become a compelling therapeutic target to counteract the impact of oncoproteins that drive oncogenesis in various cancers. The "ubiquitin code" arises from the programmed ubiquitin linkage and branching of these chains, which regulates the fate of many cellular proteins and modulates the functions of different pathways [9]. In numerous cases, ubiquitin modification serves as a tag that directs damaged or improperly folded proteins for degradation by the ubiquitin-proteasome system (UPS) [10]. Deubiquitinating enzymes (DUBs) are a type of cysteine protease that plays a crucial role in the maintenance of the ubiquitin pool within cells by reversing the process of ubiquitination. The DUB family comprises nearly 100 enzymes, all of which are involved in removing ubiquitin from various protein substrates [11]. There are five main families of DUBs, each with its own unique characteristics: ubiquitin-specific proteases (USPs), ovarian tumor proteases (OTUs), ubiquitin C-terminal hydrolases (UCHs), Machado-Joseph disease protein domain proteases (MJDs), and Jab1/Mov34/Mpr1 Pad1 N-terminal + MPN + (JAMM) motif proteases [12]. The USP subfamily, the largest group of DUBs, is implicated in stabilizing numerous oncoproteins through deubiquitinating various target proteins in different types of cancer. By disrupting the oncogenic network of these proteins, the USP subfamily plays a significant role in cancer progression. Many USPs, including USP2, USP7, USP10, USP22, USP44, and USP9X, have been extensively studied about their role in different types of cancer, which has led to the development of inhibitors for USP1, USP7, USP9X, and USP14. These inhibitors have great potential in the treatment of various cancers [13]. USP37 (Ubiquitin specific peptidase 37) is one of the recently characterized DUB, first described by *Huang *et al.in 2011 [14], and its role in cancer development has been explored in a few studies which have come up in the last decade. USP37 has been shown to interact and deubiquitinate substrates like cyclin A [14], 14-3-3γ [15], HIF2α [16], GLI1 [17], CDT1 [18, 19], PLZF/RARα [20], BLM [21] which are not recognized by other DUBs. The role of USP37 in promoting tumorigenesis and how it regulates different substrates has been discussed in a recent review by our group, highlighting the importance of targeting USP37 for pharmacological intervention [22].

CDT1 is another protein that is intricately tied to DNA replication fork movement and is recruited to replication fork origin and aids in loading of Mini chromosome maintenance complex MCM2-7, and its levels are controlled by ubiquitin machinery in response to DNA damage. Studies have demonstrated that USP37 plays a crucial role in the stabilization of phosphorylated CDT1 through its deubiquitination. When USP37 is depleted, there is a delay in the progression of replication forks and an increase in the percentage of fork firing. These findings suggest that USP37 mediates an additional layer of proteins involved in regulating replication fork firing [18]. In a recent collaborative study, we found that depletion of USP37 in cells results in aberrant DNA replication, as evidenced by altered S phase kinetics and accumulation of DNA damage foci. Subsequent investigations revealed that USP37 is responsible for the deubiquitination of CHK1, a protein whose destabilization occurs in the absence of USP37. These findings indicate that USP37 is involved in regulating replication stress tolerance in different cellular models, including U20S osteosarcoma cells [23]. Another study in lung cancer suggested that USP37 has been shown to directly deubiquitinate MYC and block its degradation [24], and MYC expression has been associated with poor patient prognosis in osteosarcoma [8]. We hypothesize that USP37 may be a potential contributor to poor prognosis in MYC-driven cancers such as osteosarcoma. This is because USP37 is believed to promote tolerance to replication stress by stabilizing replication forks and multiple oncoproteins like CHK1 which are involved in regulating replication stress as shown in our recent study [23], thereby reducing the effectiveness of therapeutic approaches in treating osteosarcoma. In continuation to our previous study, we have provided evidence here that USP37 interacts with PCNA, which is a critical protein in replication fork movement, by functioning as a sliding clamp loader, thereby regulating replication stress tolerance in osteosarcoma cells.

## Methods

### Cell lines and other reagents

MG-63 cell line was obtained from The National Centre for Cell Science, Pune, India, while MCF10A (EP-CL-0525) and U2OS (EP-CL-0236) cells were obtained from Elabiosciences. Details of other reagents and antibodies used are provided in Additional file 2: Table S1.

### Cell culture

Osteosarcoma U2OS and MG63 cells were cultured in Dulbecco’s modified Eagle’s medium (DMEM; Gibco, Thermo Fisher Scientific) supplemented with 10% FBS (Gibco, Thermo Fisher Scientific) and 1% Pen Strep (GIBCO). MCF10A cells was cultured in DMEM/F12(EP-CM-L0113) + 5% HS + 20 ng/ml EGF + 0.5 μg/ml Hydrocortisone + 10 μg/ml Insulin + 1% NEAA(EP-CM-L0463) + 1% Pen/Strep (GIBCO). All cells were incubated at 37 ℃ and 5% CO2 and periodically tested for mycoplasma contamination.

### Transfections and treatments

siRNA transfections were performed using Lipofectamine RNA iMAX (Thermo Fisher Scientific) according to the manufacturer's protocol. Two sequences of siRNAs have been used in the current study; one is Mission Si RNA USP37 (Sigma Aldrich) with the target sequence; TGAGGTTCAGCACTCCATCATTTGTAAAGCATGTGGAGAGATTATCCCCAAAAGAGAACAGTTTAATGACCTCTCTATTGACCTTCCTCGTAGGAAAAAACCACTCCCTCCTCGTTCAATTCAAGATTCTCTTGATCTTTTCTTTAGGGCCGAAGAACTGGAGTATTCTTGTGAGAAGTGTGGTGGGAAGTGTGCTCTTGTCAGGCACAAATTTAACAGGCTTCCTAGGGTCCTCATTCTCCATTTGAAACGATATAGCTTCAATGTGGCTCTCTCGCTTAACAATAAGATTGGGCAGCAAGTCATCATTCCAAGATACCTGACCCTGTCATCTCATTGCACTGAAAATACAAAACCACCTTTTACCCTTGGTTGGAGTGCACATATGGCAATTTCTAGACCATTGAAAGCCTCTCAAATGGTGAATTCCTGCA and other is USP37 siRNA (sc-76845) (Santacruz Biotechnology). Scrambled siRNA (SC 37007, Santacruz Biotechnology) was used a control. Myc-USP37 and USP37 C50A plasmids were gift from Summers Lab, Ohio State University, Columbus, Ohio, USA, and have been described previously [23]; HA-Ubiquitin (Plasmid #18712) plasmid was purchased from addgene repository where it was deposited by Edward Yeh lab. Plasmid transfections were performed using Lipofectamine 3000 and RNA iMAX (Thermofisher) according to the manufacturer's protocol.

### Gene expression profiling interactive analysis 2 (GEPIA2)

Osteosarcoma RNA sequencing data of tumor and normal tissues was analyzed from the Cancer Genome Atlas (TCGA) datasets using GEPIA2 (http://gepia2.cancer-pku.cn/). We performed a survival analysis of USP37 expression from the survival map module in the TCGA sarcoma dataset.

### Transcriptome analysis (RNA sequencing)

RNA sequencing via the illumina platform was performed based on the mechanism of SBS (sequencing by synthesis) to understand RNA transcriptional activity and identification of genes differentially expressed in distant cell populations. Different cell populations and phenotypes are: Wild Type untreated cells(WT, UU1), USP37 overexpressed condition (OE, UU2), USP37 knockout condition (KO, UU3). Original image data file from high-throughput sequencing platforms (Illumina) is transformed to sequenced reads (called Raw Data or Raw Reads) by CASAVA base recognition (Base Calling). Raw data are stored in FASTQ (fq) format files. The statistically significant differentially expressed genes of ± 1.3 log twofold change were used for analysis. Sequencing flow is shown in Additional file 1: Figure S1.

### Venn diagram and heat map generation

Overexpressed and Knockdown of USP37 phenotypes of U2OS cells were used for generating the Venn diagram and Heat map. For Venn diagram generation, the Venny 2.1.0 tool was used [25]. The heat map for both phenotypes was made in Microsoft excel using the conditional formatting option ontologies and pathways. The gene ontology and pathways for the statistical DEGs identified by the whole RNA sequencing for both OE and KO phenotypes were then generated using the Cluego plugin of the Cytoscape software [26, 27].The default parameter was used for generating the gene ontologies (Biological process, Cellular component and Molecular function) along with the Reactome and KEGG pathways.

### Immunoblotting

Cellular extracts were generated in EBC lysis buffer (50 mM Tris (pH8.0), 120 mM NaCl, 0.5% Nonidet P-40, 1 mM DTT, and protease and phosphatase inhibitor tablets) (Thermo Fisher USA). Protein concentration was quantified by Pierce BCA assay (Thermo Fisher, USA), and samples were prepared by boiling them in Laemmli buffer for 5 min. Equal amounts of whole cell lysates were resolved by hand-cast SDS-PAGE and transferred to PVDF membranes (Millipore). All blocking and primary antibody steps were performed in 5% BSA diluted in TBST (137 mM NaCl, 2.7 mM KCl, 25 mM Tris pH 7.4, 1% Tween-20). All primary antibody incubations were performed with shaking at 4℃ overnight. After the transfer of the separated proteins to PVDF membranes, the membranes were probed with the following primary antibodies: USP37 (dilution 1:1000; Elabsciences), β -actin (dilution 1:10,000; Sigma, MO, USA), γ-H2AX (at dilution 1:1000; from Santa Cruz). After overnight incubation, membranes were extensively washed and then incubated with either secondary goat anti-mouse/rabbit HRP-conjugated antibodies (Cell signalling technology). Separated protein bands were visualized using chemiluminescence HRP substrate (Bio-Rad, CA, USA), and the membranes were imaged and analyzed using the Chemi Doc™ imaging system from Bio-Rad (CA, USA).

### Colony formation assay

U2OS Cells were transfected with si-RNA and indicated plasmid using Lipofectamine RNAiMAX (ThermoFisher) and lipofectamine 3000(ThermoFisher), respectively. Cells were trypsinized, and 500 cells/well were seeded in 6 well plates. Cells were allowed to grow for two weeks until the colonies could be observed. The colonies were fixed with 4% paraformaldehyde for 30 min and stained with 0.4% crystal violet. Cells were washed with 1 × PBS to remove excess dye. The plate was left to air dry, and the colonies were counted using ImageJ software (National Institutes of Health, USA).

### Cytoplasmic and nuclear fractionation

Cytoplasmic and nuclear extraction was performed using NE-PER™ Nuclear and Cytoplasmic Extraction Reagents (Thermofisher Cat no: 78833) as per manufacturer protocol.

### RNA-isolation and quantitative real-time PCR (qRT-PCR)

Total RNA was extracted using the Trizol method and reversed transcribed into cDNA by Verso cDNA Synthesis Kit** (**AB1453A). Real-time PCR was performed on LightCycler 480 (Roche) with The LightCycler^®^ 480 SYBR Green I Master. 2^−ΔΔCT^ method was used for relative quantification. Beta-actin RNA was used for data normalization. All primer sequences of qRT-PCR are listed below: *USP37*: Forward primer: TGGCTCTCTCGCTTAACA; Reverse primer: TGCACTCCAACCAAGGGTAA *Beta Actin*: Forward primer: CTGCCGTTTTCCGTAGGACT Reverse primer: ACCTACACCCACAACACTGTC.

### Immunofluorescence microscopy

The U2OS and MG63 cells were allowed to grow for 24 h in chambered slides (SPL Lifesciences cat no 30104), then treated with 300 µM HUfor 24 h. In another set, U2OS cells were transfected with control siRNA and si-USP37 for 24 h, then plated on chambered slides for 24 h, and then treated with 300 µM hydroxy urea (HU) for 24 h. After that, cells were washed in 1X PBS, fixed with 4% paraformaldehyde for 20 min at RT, and permeabilized with 0.1% TritonX-100 for 3 min at room temperature. The cells were then washed in PBS and incubated with an anti-USP37 antibody (Elabsciences) at a 1:200 dilution, anti- γ-H2AX (dilution 1:1000; Santa Cruz), anti-53BP1(Dilution 1:1000; Santa Cruz). And anti-RPA (Dilution 1:1000: from SantaCruz). After extensive washing, a goat anti-rabbit Alexa-488 secondary antibody (1:400 dilution; Applied Biosciences) was added for 45 min at 4 °C. Finally, the cells were rinsed with 1X PBS and counter-stained with DAPI (Fluoroshield™, Sigma, MO, USA) for 2 min. For Immunofluorescence microscopy, the fixed and stained cells were mounted with the Fluorescent Mounting Medium (Dako, Glostrup, USA) and viewed with a 40/60 × objective using Leica DM6 Microscope (Wetzlar, Germany).

### Immunoprecipitation

Cells were transfected with the indicated plasmids and lysed in IP lysis buffer 48 h after transfection. Following lysis, protein estimation was done using BCA Kit (Pierce USA). 100 µg lysate was taken and cleared out using Protein A/G agarose beads (Sigma Aldrich) by incubating them in a spin rotator for 1 h., following which lysate was centrifuged to remove the Protein A/G agarose beads. About 1 μg of the indicated antibody i.e., anti-USP37 (Novus Biologicals, #cat NB110-40709) or Anti PCNA Antibody, was mixed with lysate at 4 °C overnight. Protein A/G beads were then added and incubated at 4 °C for 1 h. to capture the antibody-antigen complex. Beads were washed three times with IP lysis buffer, and proteins were eluted by boiling beads in Laemmli buffer for 5 min and visualized by immunoblotting using respective antibodies as indicated.

### Ubiquitination/deubiquitination assay

U20S cells were transfected with the indicated plasmids. After 48 h., the cells were treated with 100 µM HU and 100 μM MG132(Sigma Aldrich) for 4 h. and then harvested. The cell suspension was lysed in 6 M guanidine–HCl, 100 mM Na2HP04–NaH2P04, 10 mM Tris–HCl, pH 8.0, 5 mM imidazole, 10 mM β-mercaptoethanol, and proteins were captured using Protein A/G Agarose (Sigma) for 4 h. at room temperature. The beads were washed sequentially in lysis buffer (without imidazole), buffer A, pH 8 (8 M urea, 100 mM Na2HP04–NaH2P04, 10 mM Tris–HCl,pH 8.0, and 10 mM β-mercaptoethanol), buffer A pH 6.3 + 0.2% Triton X-100, and buffer A pH 6.3 + 0.1% Triton X-100, and eluted by boiling in Laemmli buffer containing 200 mM imidazole. About 10% of the sample was used to prepare inputs. Pull-down eluates and inputs were separated on SDS-PAGE gels and analyzed by immunoblotting.

### Molecular modeling and MD simulations of USP37-PCNA and USP37-PCNA-DNA Complex

The full-length structure model of human USP37 was generated using the I-TASSER server [28–30]. The structure model was energy minimized and subjected to 100 ns MD simulations using Gromacs-5.0.7 [31]. The structure model was validated using Procheck Server [32]. To generate a functional complex of USP37-PCNA-DNA, first, a trimeric model of the human PCNA-DNA complex was built using the Cryo EM structure of yeast PCNA-DNA complex as the template (PDB ID 6KNB) [33]. The structure complex was energy minimized before performing protein–protein docking with USP37. The docking of the USP37 pleckstrin domain (PH) with the trimeric PCNA-DNA complex was performed using Cluspro Server using standard protocol without defining the binding site [34, 35]. A total of 179 Clusters were obtained, and the best-docked cluster with the lowest energy score of − 1585.8 and the center-weighted score of -1389.8 was selected for further 100 ns MD simulations using Gromacs-5.0.7 [31]. To perform MD, a 10 Å cubic box (in each direction from complex atoms) [36] solvated with the TIP3P water model was generated and neutralized using Na^+^ions [37]. Further, the system was subjected to energy minimization using the steepest descent algorithm with the convergent criteria of < 1000.0 kJ/mol/nm. The forcefields AMBER 99SB-ILDN (protein) and AMBER94 (DNA) were used for the MD run. The DNA atoms were restrained and equilibrated at 300 K temperature and 1 atm pressure using the Berendsen thermostat and Parrinello-Rahman barostat in Periodic Boundary Conditions. The 100 ns MD run was performed using the Leapfrog Dynamics integrator and velocity integration at 1 fs step size. The LINCS algorithm was used for constraining all the bonds [38]. The PME (Particle Mesh Ewald) and Varlet scheme algorithms were used for calculating the long-range electrostatic forces and searching the short-range forces, respectively [39–42].

### DNA replication restart assay

Exponentially growing cells were pulse-labelled with 50 mM5-chlorodeoxyuridine (CldU) for 30 min, washed three times with PBS, treated with 2 mM hydroxyurea (HU) for 2 h., washed three times with PBS, incubated again in fresh medium containing 50 mM5-iododeoxyuridine (IdU) for 60 min, and then washed three times in PBS. DNA fiber spreads were made by a modification of a procedure described previously [43]. Briefly, cells labeled with IdU and CldU were mixed with unlabelled cells at a ratio of 1:10, and 2 µl cell suspensions were dropped onto a glass slide and then mixed with 20 µl hypotonic lysis solution 10 mM Tris-HCl (pH 7.4), 2.5 mM MgCl2, 1 mM phenylmethylsulphonyl fluoride (PMSF), and 0.5% Nonidet P-40) for 8 min. Air-dried slides were fixed, washed with 1 × PBS, blocked with 5% bovine serum albumin (BSA) for 15 min, and incubated with primary antibodies against IdU and CldU. Rat anti-IdU monoclonal antibody [MAb] (1:150 dilution, Abcam) and mouse anti-CldU MAb (1:150 dilution; BD) and secondary antibodies anti-rat Alexa Fluor 488-conjugated [1:150 dilution] and anti-mouse Alexa Fluor 568-conjugated [1:200 dilution] antibodies for 1 h. each. Slides were washed with 1X PBS with 0.1% Triton X-100 and mounted with Vectashield mounting medium without 4 = ,6-diamidino-2-phenylindole (DAPI). ImageJ software was used to analyze the DNA fibers. For each data set, about 300 fibers were counted for stalled forks, new origins, or elongated forks, and the results were summarized in bar graphs.

### Immunohistochemistry

All tissue sections were first deparaffinized with xylene, followed by acetone dehydration and ethanol rehydration. Following heat-induced antigen retrieval of sections in a recommended buffer, i.e., (Tris-EDTA, pH-8) for 30 min at 100 °C. Sections were then incubated with blocking buffer (H_2_O_2_) for 20 min to quench the endogenous peroxidase activity. (Ultra Vision Quanto Detection System HRP DAB #cat.TL-060-QHD). To clear out non-specific binding, blocking was done using a blocking buffer for 15 min. Then the sections were incubated with anti-USP37 (cat. No. E-AB-19606 1:100) or anti-PCNA (Cat. No. 2978 for IHC; 1:200) primary Ab at 4 °C overnight and then with HRP-conjugated goat anti-rabbit IgG (1:200) secondary Ab. Finally, the sections were reacted with diaminobenzidine and counterstained with hematoxylin. The IHC-stained sections were assessed under a microscope by two histopathologists. The semi-quantitative IHC scores of USP37 and PCNA were recorded, ranging from low expression (minimum score) and intermediate expression to high expression (maximum score).

### Statistical analysis

Statistical analyses were performed with GraphPad Prism Software using an unpaired Student's *t* test one- or two-way ANOVA with post-tests, as indicated.

## Results

### Elevated expression of USP37 correlates with poor prognosis of osteosarcoma patients

**Fig. 1:**
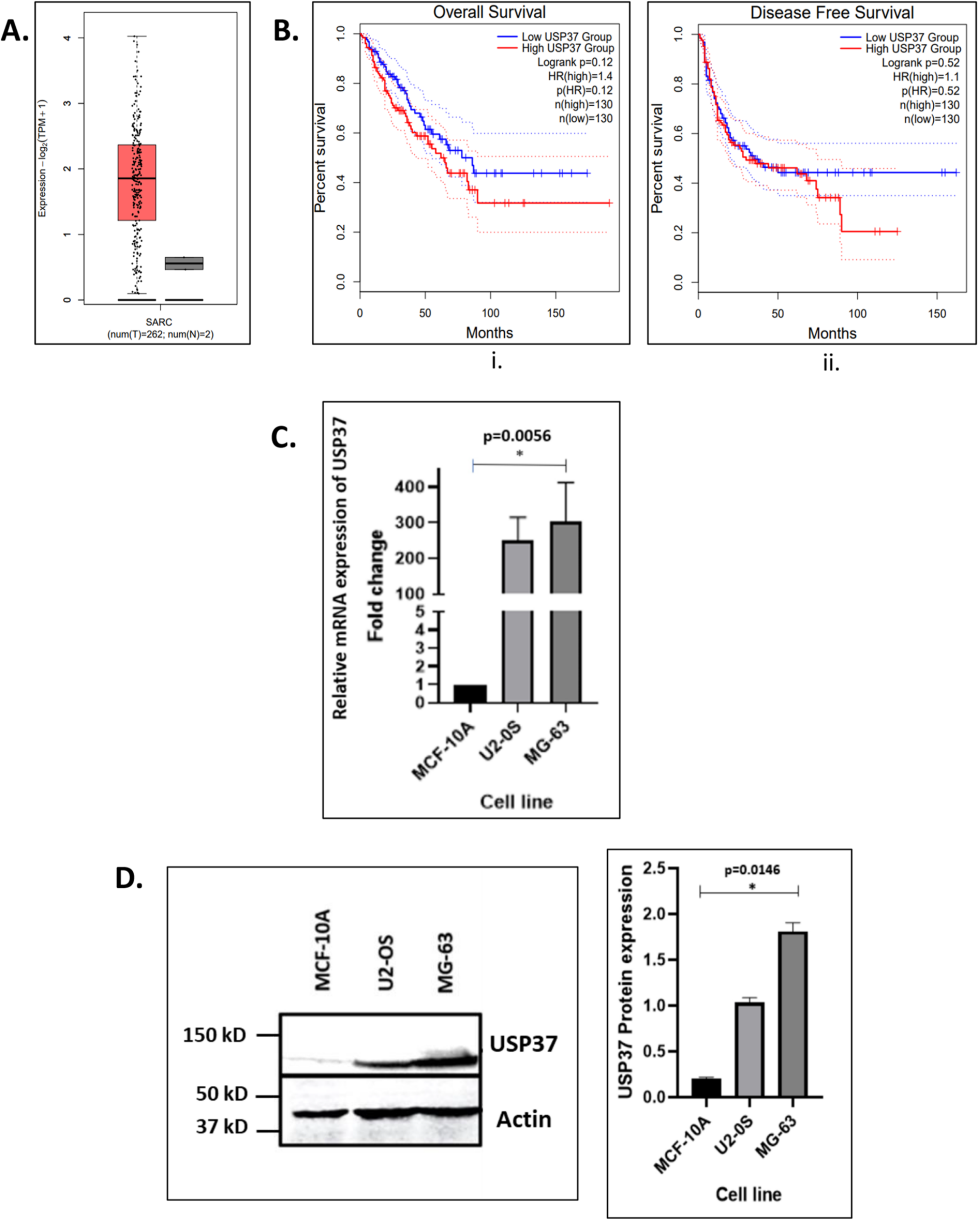
Elevated expression of USP37 correlated with poor prognosis of osteosarcoma patients. **A** TCGA database analysis of USP37 expression in sarcoma versus normal tissue. **B** Median USP37 expression correlation with (i). overall survival (OS) in sarcomas. Median USP37 expression correlation with (ii). disease-free survival (DFS) in sarcomas. **C.** Relative mRNA expression of USP37 in Osteosarcoma cell lines (U2OS and MG63) as compared to non-transformed cell line (MCF-10A). **D**. Relative protein expression of USP37 in Osteosarcoma cell lines (U2OS and MG63) as compared to non-transformed cell line (MCF-10A)

USP37 is overexpressed and correlated with various hallmarks like angiogenesis, apoptosis resistance, DNA replication, etc. [22]. Our data analysis using TCGA and GTEx through GEPIA2 showed significant overexpression of USP37 in the osteosarcoma patients(n = 262) as compared to benign controls (Fig. 1A, Additional file 1: Fig S2A, B). Furthermore, we observed significant correlation of USP37 overexpression with overall survival (OS) and Disease Free Survival (DFS) in osteosarcoma patients (Fig. 1Bi, Bii). Our Kaplan -Meier curve analysis showed that osteosarcoma patients with high USP37 transcript levels have reduced OS and DFS as compared to a patient with low USP37 levels (Fig. 1Bi, Bii). Similar to patient data, increased expression of USP37 was seen in osteosarcoma cell lines U2OS and MG-63 both at both mRNA (Fig. 1C) and protein level (Fig. 1D) as compared to non-transformed breast epithelial MCF10A cells [46, 47]. More importantly, increased expression of USP37 was seen in MG-63 cells as compared to U20S cells which are characterized by highly invasive potential and faster growth as compared to less invasive and stationary cell line MG-63 [44, 45].

After a correlation of OS and DFS was seen with the expression of USP37, we modulated the expression of USP37 by overexpression and depletion of USP37 to identify its molecular targets in osteosarcoma especially pertaining to replication stress. (Additional file 1: Figure S2C, D) as we have recently shown that USP37 regulates tolerance of replication stress by enhancing CHK1 activity so in order to gain insight into genes that correlated with knock down or overexpression of USP37, we proceeded with exome sequencing analysis in osteosarcoma cells overexpressing USP37 or in cells in which USP37 was depleted.

### Alternation in expression of USP37 leads to activation and repression of distinct set of genes involved in different pathways

**Fig. 2:**
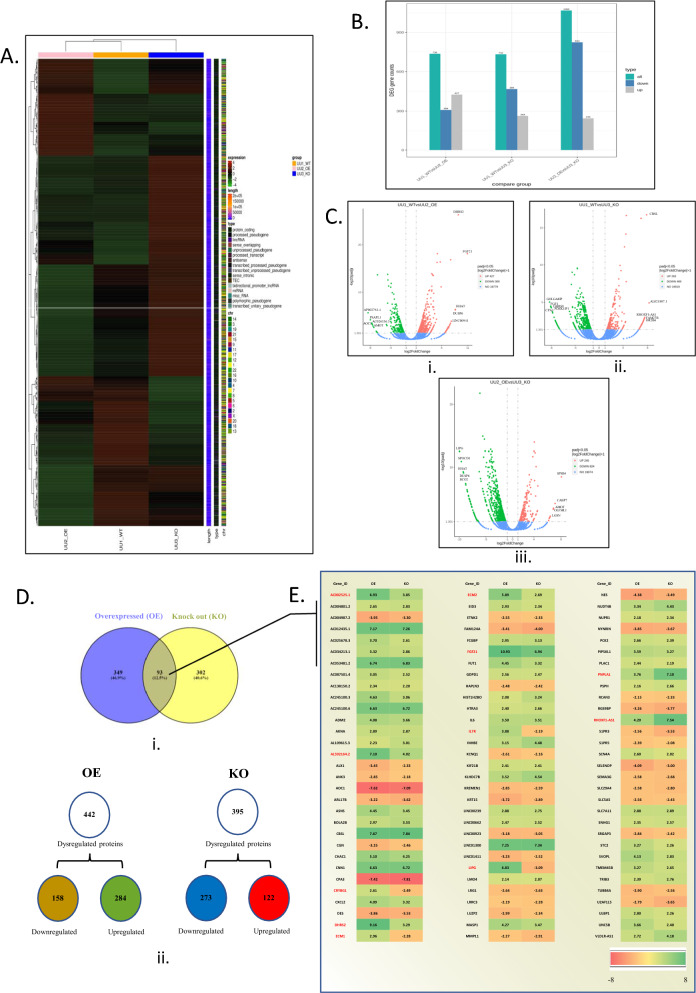
Alternation in the expression of USP37 leads to activation and repression of a distinct set of genes involved in different pathways: Whole cell RNA sequencing of U2OS cells before and after USP37 overexpression and Knock down. **A** The Heat map representation of all 3 phenotypes (WT or untreated, OE and KO) along with RNA type and chromosomal position of the DEGs with the ± 1.301 log_2_ fold change cut off. **B** Histogram showing the distribution of DEGs in 3 phenotypes (i)OE vs WT, (ii)KD vs WT and (iii)OE vs KD. **C** Volcano plots of the DEG in 3 different phenotypes with ± 1.301 log_2_ cut off. Distribution of RNA/genes vis whole RNA sequencing of U2OS cells after USP37 Overexpression and Knock down**. D** The Venn diagram shows the ± 2 Log_2_ fold statistically significant genes/RNA of U2OS cells as compared to untreated cells after USP37 overexpression and Knock down. **E** In addition, the Heat map shows the comparison of statistically significant genes found in both USP37 overexpression and KO phenotypes when compared to untreated cells

After analyzing the TCGA data and observing elevated USP37 expression in the osteosarcoma cohort, we performed overexpression (OE) and knockdown (KD) experiments of USP37 using Myc-tagged USP37 plasmid [23], and si USP37 in the U2OS cell line. Subsequently, RNA sequencing analysis was performed to identify the potential pathways and factors regulated by USP37. We generated a heat map showing the significant changes in gene sets among all three phenotypes wild type (WT), Overexpressed (OE) and knock out (KO), based on expression, length, type, and chromosome (Fig. 2A). Differentially Expressed Genes (DEGs) were identified using a ± 1.301 log2 fold change cut-off and presented in a histogram (Fig. 2B). We generated comparative volcano plots among all phenotypes (Fig. 2Ci), which showed 427 genes upregulated and 309 genes downregulated in the overexpressed phenotype compared to wild type (Fig. 2Cii), 263 genes upregulated and 469 genes downregulated in the KO phenotype compared to wild type, and 245 genes upregulated and 824 genes downregulated when comparing OE and KO phenotypes (Fig. 2Ciii). To further analyze the dysregulated gene set in osteosarcoma cells after USP37 overexpression and knockdown, we used a ± 2 Log2 fold cut-off and identified a total of 349 genes dysregulated in the OE condition and 302 genes dysregulated in the KO condition compared to wild type (Fig. 2Di) [25]. Ninety-three genes were commonly dysregulated in both phenotypes compared to WT. A detailed heat map showing all 93 commonly dysregulated genes is provided in Fig. 2E.

**Fig. 3:**
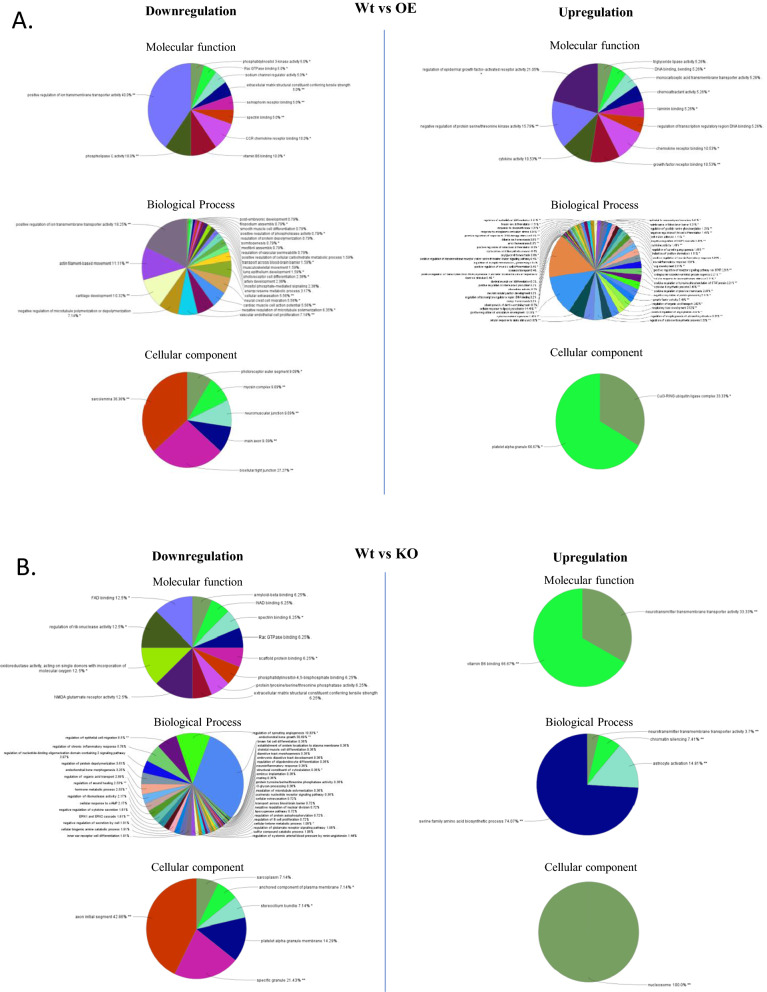
Alternation in expression of USP37 leads to activation and repression of distinct set of genes involved in different pathways. **A** Gene ontologies of downregulated and upregulated genes after USP37 overexpression in U2OS cells by cluego. **B** Gene ontologies of downregulated and upregulated genes after USP37 KO in U2OS cells by cluego

After identifying the commonly deregulated genes in both USP37 OE and KO conditions compared to WT (Fig. 2Di), we analyzed the patterns of deregulation and found that 158 genes were downregulated and 284 genes were upregulated in the OE condition, while in the USP37 KO condition (Fig. 2Dii), 273 genes were downregulated and 122 genes were upregulated (Fig. 2Dii). We generated heat maps and tables listing the statistically significant deregulated genes/RNAs in U2OS cells after USP37 OE (Additional file 1: Figure S3A), and KD (Additional file 1: Figure S3B). Additional file 3: Table. S2 (WT vs OE), Additional file 4: Table S3 (WT vs KO), Additional file 5: Table S4 (OE vs KO) lists the deregulated gene in different conditions. We then used ClueGO and Cytoscape [26, 27] to perform gene ontology analysis and identify the involvement of these deregulated genes in molecular functioning, biological processes, and cellular components across all phenotypes (Fig. 3A, B). Pie charts were generated to represent the results. Additionally, we used ClueGO and Cytoscape to analyze the involvement of these deregulated genes in different pathways, which were represented in the form of KEGG and Reactome pathways (Additional file 1: Figure. S4A, B). Furthermore, we created string networks to identify upregulated and downregulated genes after USP37 OE and KO with a confidence level of 0.4 (Additional file 1: Figure S5). We also constructed the USP37 interaction map by Biogrid and visualized Interactions of USP37 with and without High throughput screening (Additional file 1: Figure S6). Overall, our exome sequencing data suggests that USP37 regulates metabolic pathways, cell cycle progression, DNA damage response, inflammation, cell–matrix adhesion, epithelial to mesenchymal transition, and immune response.

### Overexpression of USP37 leads to enhanced survival in response to replication stress while its depletion leads to reduces survival in osteosarcoma cells with increased accumulation of USP37 in cytoplasm

**Fig. 4:**
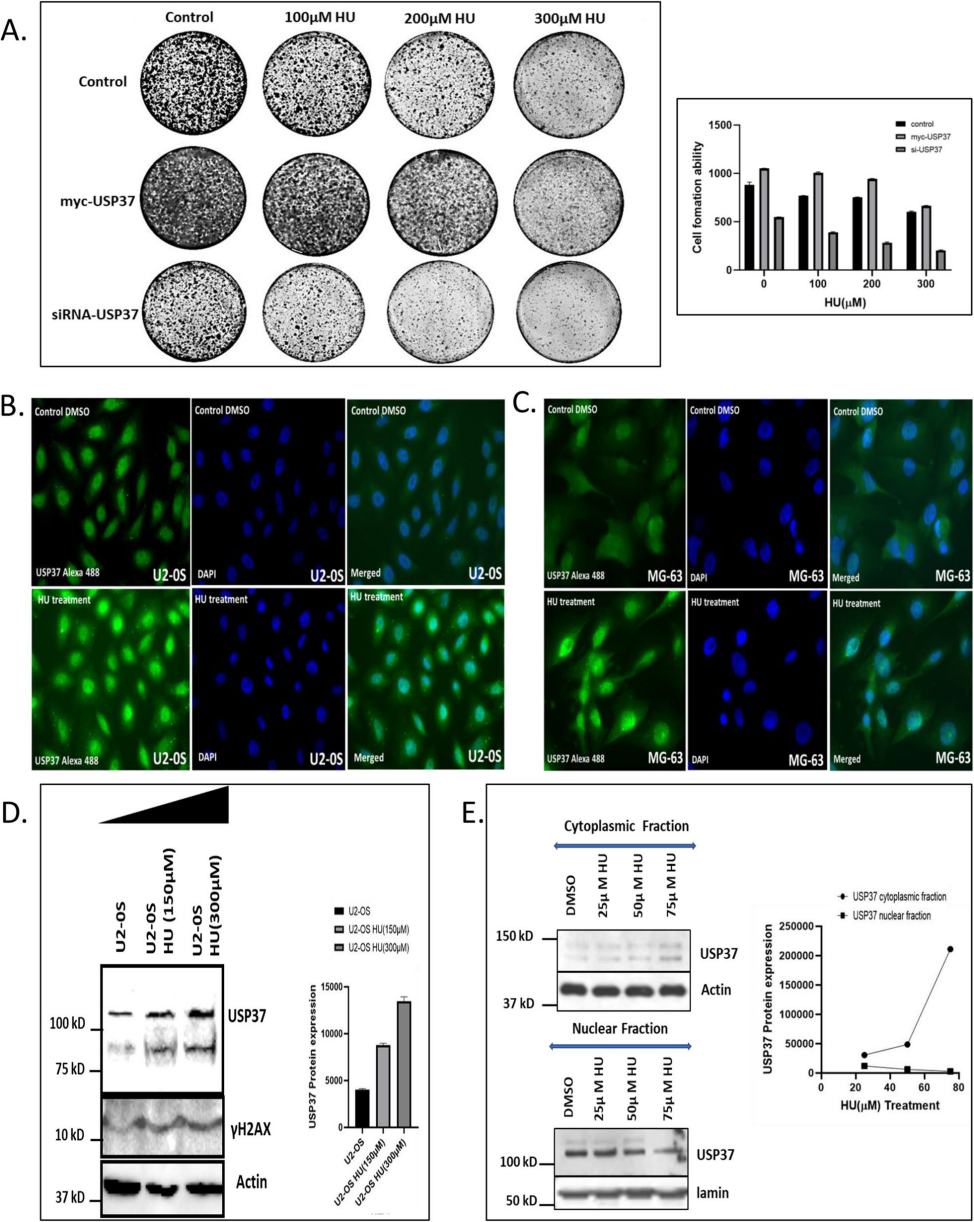
Overexpression of USP37 leads to enhanced survival in response to replication stress while its depletion leads to reduced survival in osteosarcoma cells with increased accumulation of USP37 in cytoplasm. **A** The clonogenic potential was examined by Colony formation assay. U2OS osteosarcoma cells were transfected with Myc USP37 for overexpressing USP37 while SiRNA was used to reduce expression of USP37, following which cells were treated with different concentrationsn of HU (Hydroxy Urea) for 24 h., then washed and fresh media was added. Subsequently colonies were allowed to develop for 7 days. **B** Osteosarcoma cell U20S cells were treated with 300 µM HU for 24 h and processed for IF to see USP37 expression. **C** Osteosarcoma cell line MG-63 was treated with 300 µM HU for 24 h. and processed for IF to see USP37 expression. **D** Western blotting to see the expression of USP37 and corresponding DNA damage marker ϒH2AX after induction of replication stress in U2OS whole cell lysate. **E** Nuclear and cytoplasmic fractionation was carried out on U2OS cells after treatment by an escalating dose of HU and western blotting was done to assess the level of USP37 in the nuclear and cytoplasmic fraction

Previous studies have indicated that USP37 is involved in various pathways and regulates the cell cycle, such as cyclin A, SCF^βTrCP^/CUL1, and βTrCP [14, 48]. Recent studies have shown that USP37 is involved in oncogenesis and plays a crucial role in stabilizing oncoproteins and factors involved in cellular proliferation, such as MYC and PLZF-RARA [20, 24, 49]. In a recent study, we demonstrated that USP37 enhances CHK1 activity to promote cellular survival in response to replication stress, and its depletion leads to reduced survival [23]. To further investigate the role of USP37 in cellular survival under replication stress in osteosarcoma cells, we inhibited USP37 using si-USP37 and exposed U2OS osteosarcoma cell line to HU-mediated replication stress (100 μM, 200 μM, 300 μM HU) for 24 h. The results showed reduced survival of U2OS cells in colony formation assays after USP37 inhibition and exposure to HU-mediated replication stress, while overexpression of USP37 led to enhanced survival in response to HU (Fig. 4A), suggesting that USP37 plays a crucial role in aiding the survival of osteosarcoma cells under replication stress.

Exposure to replication stress has been shown to affect the spatial localization and levels of USP37 and its associated interacting partners. Previous studies have demonstrated that USP37 interacts with CDT1, a key protein in replication fork progression, and stabilizes its phosphorylated form [18]. We sought to investigate the effect of replication stress on USP37 in osteosarcoma cells. Upon exposure of the cells to HU-mediated replication stress and subsequent visualization using immunofluorescence, an increase in the level of USP37 protein and a change in its localization pattern were observed in both osteosarcoma cell lines **(**Fig. 4B, C**)**. These results were further supported by western blotting, which showed an increase in USP37 protein levels (Fig. 4D), and by an accumulation of DNA damage, as indicated by an increase in γH2AX fraction upon exposure to HU (Fig. 4D).

We then analyzed the cellular localization of USP37 in response to replication stress by treating osteosarcoma cells with HU and isolating the nuclear and cytoplasmic fractions. Our results showed that upon induction of replication stress, there was an increase in the cytoplasmic fraction of USP37, while there was a simultaneous decrease in the nuclear fraction, indicating the movement of USP37 from the nucleus to the cytoplasm following replication stress (Fig. 4E). This data has important implications for understanding the effects of cytotoxic therapy in osteosarcoma patients. We correlated these results with immunohistochemistry data from an archived cohort of osteosarcoma patients to further validate our findings. Additionally, this data provides insight into the spatial movement of USP37 following replication stress, which may contribute to chemoresistance through its interactions with other proteins in the cytoplasm.

### Loss of USP37 leads to accumulation of spontaneous replication stress.

**Fig. 5:**
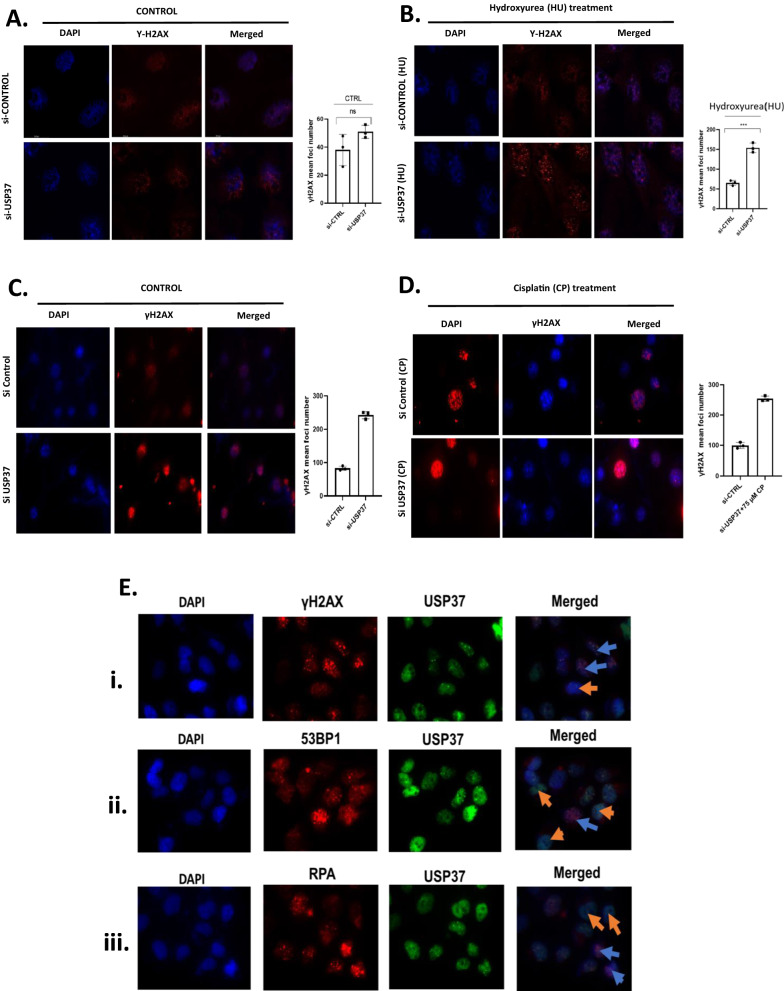
Loss of USP37 leads to accumulation of spontaneous replication stress. **A** U2OS osteosarcoma control cells and cells depleted of USP37 were stained with Anti γH2Ax antibody to assess replication stress after 48 h. **B** U2OS osteosarcoma control cells and cells depleted of USP37 were treated with 300 µM HU for 24 h and stained with Anti γH2Ax antibody to assess DNA damage response. **C** U2OS osteosarcoma control cells and cells depleted of USP37 were stained with Anti γH2Ax antibody to assess replication stress after 48 h. **D** U2OS osteosarcoma control cells and cells depleted of USP37 were treated with cisplatin and stained with Anti γH2Ax antibody to assess DNA damage response. **E** U2OS osteosarcoma cells were transfected by Myc USP37 plasmid and were treated with 300 µM HU for 24 h. and stained with i. Anti γH2AX, ii. Anti 53BP1iii. Anti RPA. Anti USP37 antibody to assess early DNA damage response in cells having a high and low expression of USP37. Blue arrows are cells with low expression of USP37, while orange arrows are cells with high expression of USP37

Recently we demonstrated that USP37 enhances CHK1 activity to promote the cellular response to replication stress [23]. Depletion of USP37 in osteosarcoma cells resulted in the accumulation of replication stress, as indicated by the spontaneous accumulation of γH2AX foci (Fig. 5A). When these depleted cells were exposed to HU for 24 h., we observed an increased accumulation of unresolved γH2AX foci (Fig. 5B). Similarly, upon exposure to 75 µM cisplatin (CP)(Sigma Aldrich), which induces DNA damage, depletion of USP37 resulted in enhanced accumulation of γH2AX foci (Fig. 5C, D). We also examined the accumulation of 53BP1 foci, a marker of double-stranded DNA breaks, and found that depletion of USP37 and exposure to HU resulted in the accumulation of unresolved 53BP1 foci (Additional file 1: Figure S7B) as well as γH2AX foci (Additional file 1: Figure S7A). To further validate these findings, we transfected U2OS osteosarcoma cells with Myc USP37 plasmid and treated them with 300 µM HU for 24 h. We then stained the cells with anti-γH2AX, anti-53BP1, anti-RPA, and anti-USP37 antibodies to assess the early DNA damage response in cells with high and low expression of USP37 upon transient transfection (Fig. 5E). We observed that cells with high expression of USP37 were able to efficiently resolve γH2AX, 53BP1, and RPA foci (marked by orange arrow) as compared to cells with low expression of USP37 (marked by blue arrow). Overall, these assays confirm that USP37 is intricately tied to DNA replication, and its depletion affects the dynamics of DNA replication.

### USP37 stabilizes PCNA (proliferating cell nuclear antigen) with and without replication stress and interacts with PCNA

**Fig. 6:**
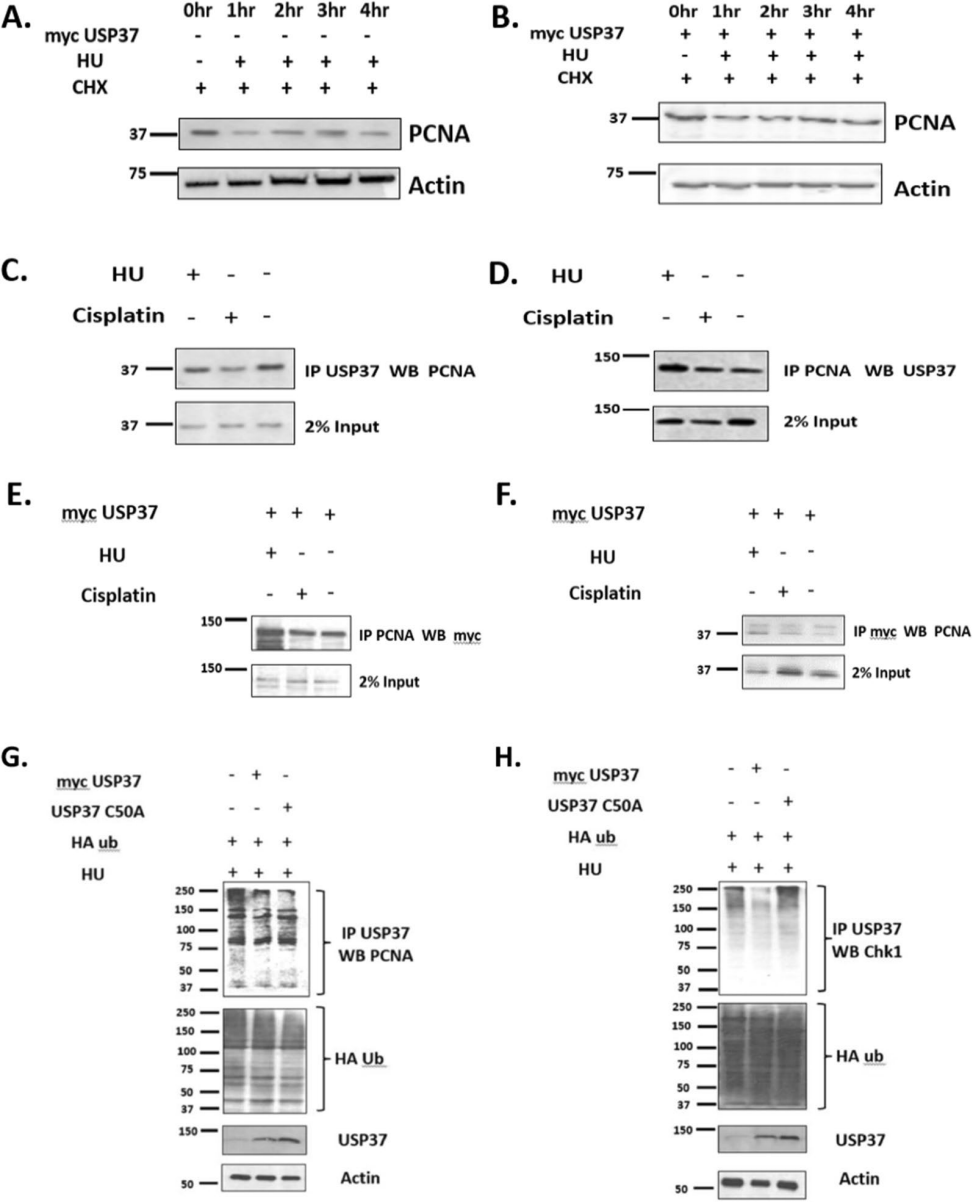
USP37 stabilizes PCNA (Proliferating Cell Nuclear Antigen) with and without replication stress and interacts with PCNA. **A** Cells were treated with Hydroxyurea (100 μM) and cycloheximide (100 μg/ml) for up to 4 h and then harvested to perform WB with Anti PCNA antibody. **B** Cells were transfected with Myc USP37 and treated with Hydroxyurea (100 μM) and cycloheximide (100 μg/ml) for upto 4 h and then harvested to perform WB with Anti PCNA antibody. **C.** Cells were treated with HU (100 μM) and Cisplatin (75 μM) for 24 h and then harvested to perform Immunoprecipitation. IP was done using an Anti-USP37 antibody while WB was done using an Anti PCNA antibody. **D** Cells were treated with HU (100 μM) and Cisplatin (75 μM) for 24 h and then harvested to perform Immunoprecipitation. IP was done using Anti PCNA antibody while WB was done using Anti USP37 antibody. **E** Cells were transfected with Myc USP37 and treated with HU (100 μM) and Cisplatin (75 μM) for 24 h and then harvested to perform Immunoprecipitation. To check the interaction of PCNA with USP37 IP was done using Anti PCNA antibody while WB was done using an anti-Myc antibody **F** Cells were transfected with Myc USP37 and treated with HU (100 μM) and Cisplatin (75 μM) for 24 h and then harvested to perform Immunoprecipitation. Reverse IP was done and capture was done using Anti Myc antibody while WB was done using anti-PCNA antibody. **G.** U20S cells were transfected with plasmids encoding HA-Ub, HA-Ub + myc USP37, HA-Ub + USP37 350A and treated with 100 μM hydroxyurea (HU) and 10 μg/ml MG132 as indicated. Lysates and ubiquitin-conjugated proteins were analyzed by immunoblotting as indicated. **H.** U20S cells were transfected with plasmids encoding HA-Ub, HA-Ub + myc USP37, HA-Ub + USP37 350A and treated with 100 μM hydroxyurea (HU) and 10 μg/ml MG132. Lysates and ubiquitin-conjugated proteins were analyzed by immunoblotting by respective antibodies as indicated

In our recent study [23], we investigated the interacting partners of USP37 at the replication fork by analyzing the proteomic profile of various cell lines [50], out of which 44 had data related to USP37 expression. We found that USP37 exhibited variable correlation with different replication factors and DNA damage proteins, including Chk1 and PCNA, which plays a crucial role in the movement of replication fork proteins as a sliding clamp loader [51, 52]. Furthermore, in our analysis, USP37 showed a stronger correlation with proteins involved in replication stress response as compared to DNA damage response. In this study, we overexpressed USP37 in U2OS osteosarcoma cells and exposed them to replication stress induced by HU. We observed increased stabilization of PCNA in cells overexpressing USP37 (Fig. 6B) compared to cells without USP37 overexpression (Fig. 6A), indicating a potential interaction between USP37 and PCNA To test this hypothesis, we treated cells with two cytotoxic agents, HU and cisplatin, and performed immunoprecipitation using anti-USP37 antibody, which was subsequently probed with anti-PCNA antibody. We observed that USP37 interacted with PCNA, and this interaction increased upon treatment with HU and cisplatin (Fig. 6C). Similarly, when we performed reverse immunoprecipitation using anti-PCNA antibody and subsequently probed with anti-USP37 antibody, we obtained similar results (Fig. 6D). We further overexpressed myc-USP37 in U2OS cells and performed antigen capture using PCNA antibody, which was subsequently probed with myc antibody, and we observed an interaction between these two proteins (Fig. 6E). These results were confirmed by reverse immunoprecipitation as well (Fig. 6F).

Deubiquitin enzymes (DUBs) can interact with PCNA and facilitate its deubiquitinylation. USP1, for example, acts as a safeguard against error-prone Translesion DNA synthesis by interacting with PCNA and promoting its deubiquitinylation [53]. Similarly, USP7 modulates UV-induced PCNA monoubiquitination by interacting with and deubiquitinating DNA polymerase eta [54]. In our study, we found that PCNA levels increase in response to replication stress in osteosarcoma cells, and we discovered that USP37 interacts with PCNA. To investigate whether USP37 directly deubiquitinates PCNA, we transfected U20S cells with plasmids encoding HA-Ub, HA-Ubmyc USP37, HA-Ub + USP37 350A, and treated them with 100 µM HU and 10 μg/ml MG132 (Fig. 6G). As a positive control, we also performed the same experiments with CHK1 deubiquitination, a known substrate of USP37 [23] (Fig. 6H). Interestingly, we observed that overexpression of either wild-type USP37 or the kinase dead mutant USP37 C50A did not cause a discernible change in PCNA ubiquitinylation (Fig. 6G). This suggests that PCNA may function as a scaffold for USP37 activity, helping to bring other key replication factors, such as CDT1, into close proximity to USP37, as previously reported [18, 19].

### Depletion of USP37 leads to alternation replication fork and increase in number of stalled fork

**Fig. 7:**
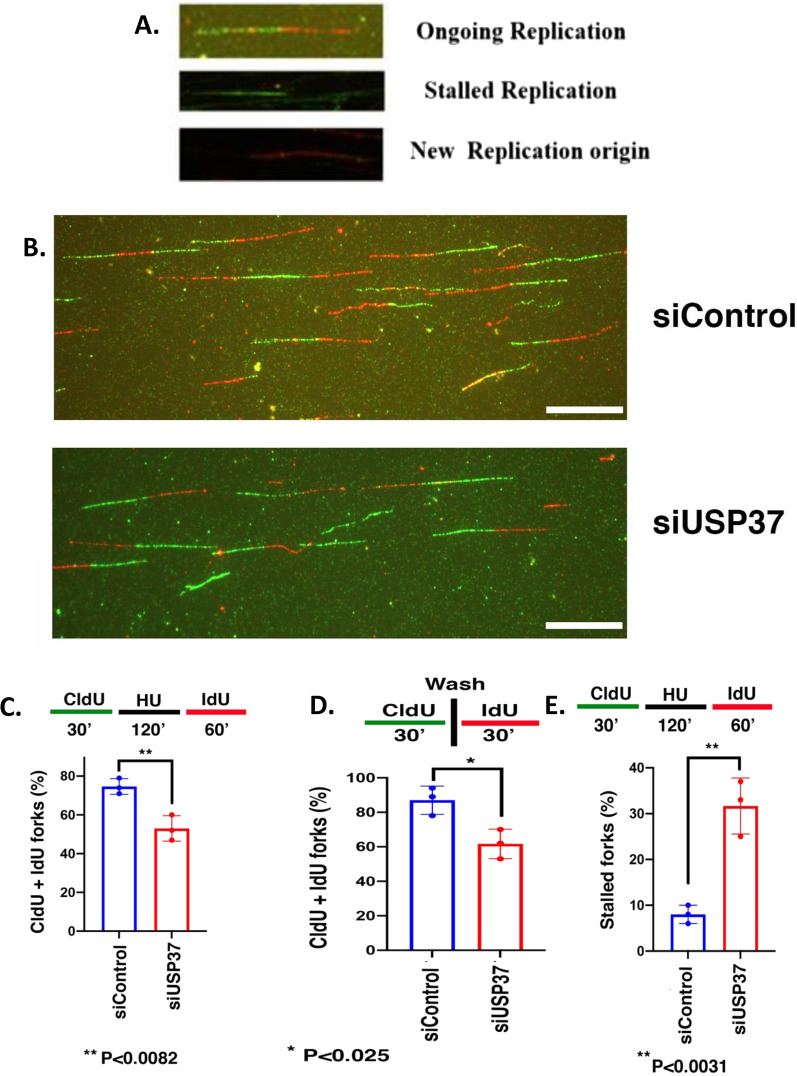
Depletion of USP37 leads to alternation in replication fork and an increase in the number of stalled fork. **A** Examples of DNA fiber tract types representing ongoing Replication, stalled Replication fork, and new origins. **B** Representative images of DNA fiber tracts in control and USP37-depleted cells after HU treatment. CldU tracts were visualized in green and IdU was visualized in red in control and USP37-depleted cells. **C** Quantitative analysis of the DNA fiber replication restarts assay without HU treatment. Bar graphs show the quantification of total tracts with both CldU and IdU labels at the fork in control and USP37 depleted Osteosarcoma cells. The experiment was performed three times, and for each time point, about 300 fibers were counted. Error bars represent the standard deviation from three independent experiments. **D** Quantitative analysis of the DNA fiber replication restarts assay with HU treatment. Bar graphs show the quantification of total tracts with both CldU and IdU labels at the fork (CldU,IdU) in control and USP37-depleted Osteosarcoma cells. The experiment was performed three times, and for each time point, about 300 fibers were counted. Error bars represent the standard deviation from three independent experiments. **E** Quantitative analysis of the replication restart assay (Percentage of the Stalled fork) after HU treatment in cells transfected with si RNA control and si-RNA USP37

Our previous studies have demonstrated that USP37 plays a crucial role in regulating replication stress and stabilizing Chk1. Additionally, USP37 has been implicated in the regulation of the Homologous recombination (HR) pathway. Specifically, USP37 is recruited to double-strand breaks (DSBs) to remove RNF168-induced ubiquitin conjugates from the BRCA1-A complex, preventing the excessive spreading of RAP80-BRCA1 from DSBs and impairing DSB repair when lost [55]. Given that USP37 depletion leads to replication stress and interacts with PCNA, we further investigated if USP37 is involved in DNA replication during replicative stress conditions compared to the unstressed state. We conducted a DNA fibre assay to evaluate replication fork movement at the single-molecule resolution. U20S osteosarcoma cells were pulse-labeled with CldU to identify cells with ongoing DNA synthesis, followed by IdU pulse-labeling. Individual DNA fibers incorporating the CldU and IdU pulses were probed with fluorescent antibodies against those analogs. Measurement of the IdU tract length distribution in DNA fibers with CldU labeling indicated much shorter lengths of replicon in USP37-depleted cells than in control cells (Fig. 7B), and a decrease in the percentage of DNA fibers with co-labelling of both CldU and IdU was observed, indicating a decrease in the number of forks in USP37-depleted cells (Fig. 7D), as previously described [56]. These results suggest that USP37 proficient cells are able to carry out more robust DNA synthesis than cells where USP37 is depleted. Moreover, since USP37 is involved in the tolerance of replication stress, we tested the effect of genotoxic stress on the movement of replication forks by exposing U20S osteosarcoma cells to replication stress after incubating cells with a first label CIdU, and then following replication restart by incubating cells with a second label IdU. Three types of DNA fiber tracts were observed based on CIdU and IdU distribution, including those with continuing elongation forks, stalled forks, and fibers in which a new DNA replication origin had fired (Fig. 7A). Exposure of osteosarcoma cells to replication stress led to a further reduction in the number of replication tracts with co-labeling of both CldU and IdU, indicating that robust replication was compromised in response to replication stress in USP37-depleted cells (Fig. 7C). Additionally, USP37 depletion and exposure to HU increased the number of stalled replication forks, where replication was unable to proceed after HU exposure (Fig. 7E). In summary, our analysis of DNA replication fork dynamics suggests that USP37 is required for efficient DNA replication fork movement by being part of the replicon complex.

### Validation of USP37 interaction with PCNA using molecular docking

**Fig. 8:**
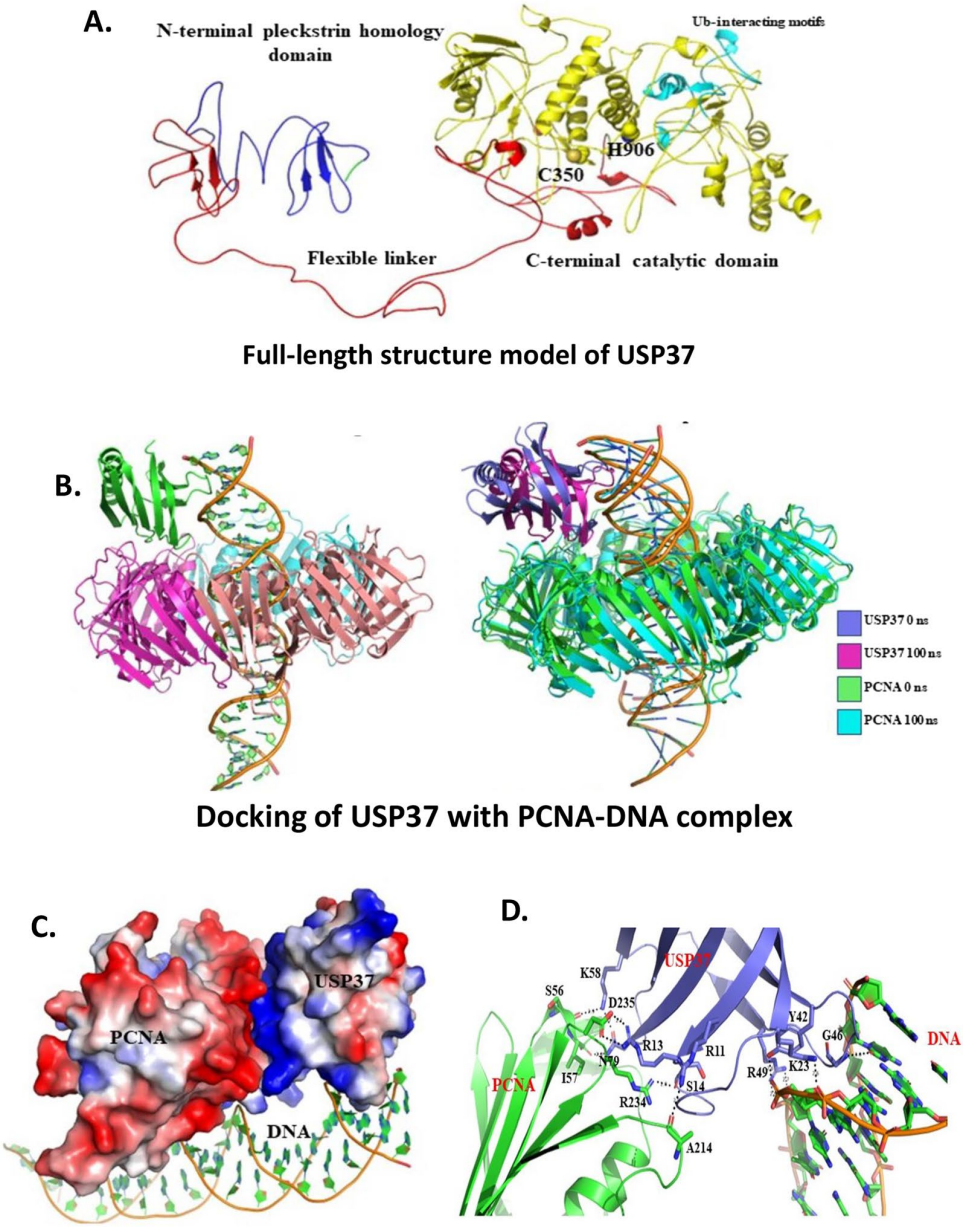
Validation of USP37 interaction with PCNA using molecular docking. **A** Structure model of full-length USP37 label with its domains. **B** USP7 PH domain docked with PCNA-DNA complex and the superimposition of trimeric PCNA-DNA-USP37 complex before and after 100 ns MD simulations. **C** The surface potential of PCNA and USP37 with DNA. **D** The interactions formed by USP37 (magenta) with PCNA (green) and DNA

To gain a deeper understanding of the relationship between USP37 and PCNA, we conducted Molecular Docking studies. The stability of the full-length structure model of human USP37 (979 residues) was verified during 100 ns MD simulations (Additional file 1: Figure S8A). The structure model consists of an N-terminal pleckstrin homology (PH) domain (4-105) and a C-terminal catalytic domain (341-951), linked by a central interdomain linker (106-340 residues) as previously described [22] (Fig. 8A.). The C-terminal catalytic domain contains three Ub-interacting motifs (UIM) (704-723, 806-825, and 828-847), which span approximately 284 amino acid residues. The N-terminal PH domain contains two destruction boxes (D) and one KEN box domain, while the central interdomain linker (central USP domain) includes a unique destruction box (D) box and unique KEN box domains that regulate the deubiquitinase (DUB) activity [22]. The active site in the C-terminal catalytic domain includes a nucleophile Cys350, Ser628, and a proton acceptor His906 residue essential for DUB activity. Our docking studies demonstrated that the USP37 PH domain interacts stably with the PCNA-DNA complex, forming five interactions (Fig. 8B). Analysis of the complex's surface potential revealed that the basic USP37 PH domain recognizes the acidic surface of PCNA and the DNA minor groove (Fig. 8C) through specific residues, including Lys23, Tyr42, Thr44, Gly46, and Arg49 (Fig. 8D) (Table 1). During the 100 ns MD simulation, the complex remained stable, as indicated by the SASA, Rg, and RMSD parameters (Additional file 1: Figure S8B) (Table 1). Out of these five interactions, the salt-bridge between Asp235- Arg13 and the hydrogen bond between Ala214-Arg13 with PCNA, and the interactions formed by Lys23, Arg49, and Thr44 with DNA minor groove persisted throughout the MD simulation (Fig. 8D) (Table 2).

**Table 1:** Interactions formed by USP37 with PCNA

S.No	PCNA	USP37	Distance (Å)	
			0 ns	100 ns
1	Ser56	Lys58	2.9	–
2	Ile57	Asn79	2.8	–
3	Ala214	Arg11	2.9	2.8
4	Arg234	Ser14	2.8	–
5	Asp235	Arg13	2.7	2.7

**Table 2:** Hydrogen bonded interaction formed by USP37 with DNA

S.No	Residues	0 ns	100 ns
1	Lys23	Present	Present
2	Tyr42	Present	Absent
3	Thr44	Present	Present
4	Gly46	Present	Absent
5	Arg49	Present	Present

### USP37 was found to be elevated in archived cohort of osteosarcoma patients and correlated with PCNA expression

**Fig. 9:**
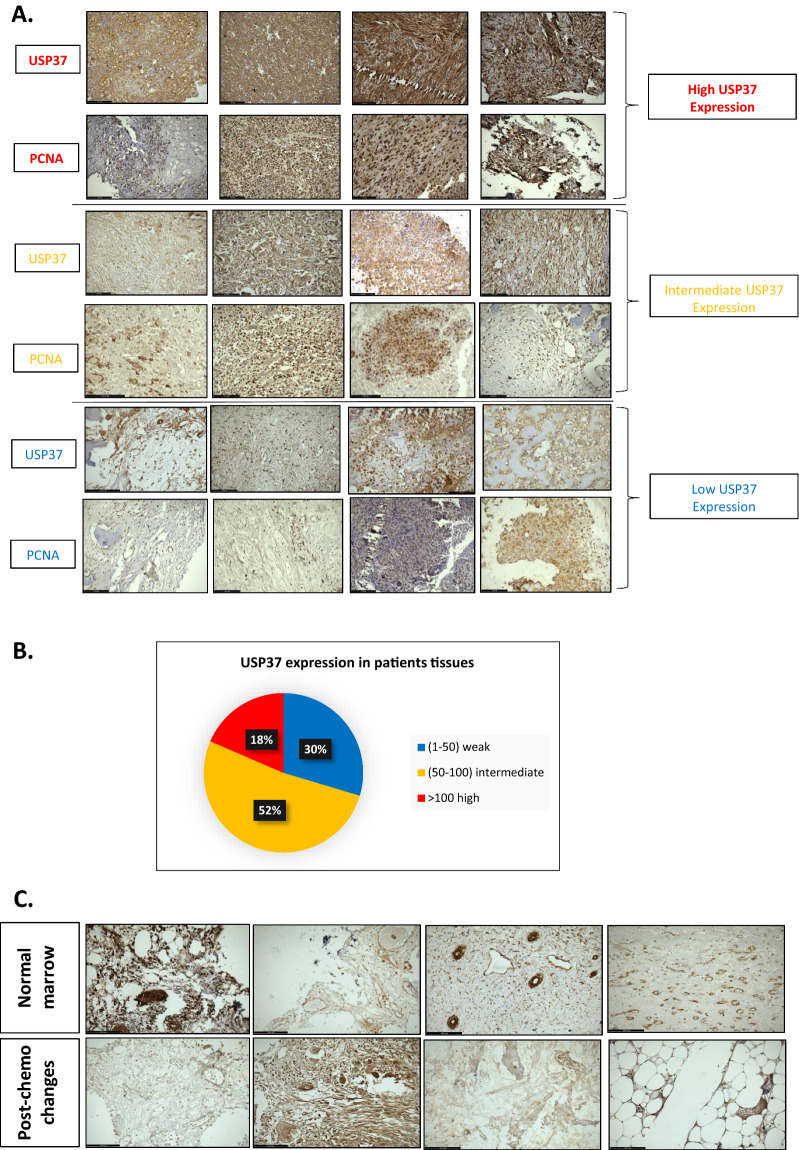
USP37 was found to be elevated in archived cohort of Osteosarcoma patients and correlated with PCNA expression. **A** Protein level of USP37 is positively correlated with PCNA expression in osteosarcoma tissue sections. The expression of USP37 and PCNA was examined by immunohistochemical (IHC)using Anti USP37 and Anti PCNA antibodies. **B** Representative data of expression profiling of USP37 in different tissue sections. **C** Representative images of USP37 and PCNA expression in normal marrow and post-chemotherapy changes induced in the tissues regarding the expression of USP37 and PCNA. The expression of USP37 and PCNA was examined by immunohistochemical (IHC) staining

The immunohistochemistry study performed on a retrospective cohort of 40 osteosarcoma patients showed that the expression of USP37 was much higher in osteosarcoma tissues compared to adjacent non-transformed tissue, and expressed in a variety of patterns which were grouped into high, medium, and intermediate expression (Fig. 9A). Approximately 52% of tissue sections showed intermediate expression of USP37 while 18% of patients showed high expression and 30% showed low expression (Fig. 9B). Clinical details of patient enrolled are provided as Additional file 6: Table S5. This suggests that USP37 may be involved in the progression of osteosarcoma. Since USP37 was found to interact with PCNA and our molecular docking data further corroborated this finding, the co-expression of PCNA was examined in the osteosarcoma tissue sections. The results indicated that USP37 protein levels positively correlated with PCNA levels, suggesting that PCNA expression can be an indicator of USP37 expression, which might indicate cell proliferative potential. Interestingly, the study also showed that USP37 expression was low in normal bone marrow, while osteosarcoma tissue sections from patients who had undergone chemotherapy-induced alterations had elevated USP37 expression (Fig. 9C). This finding suggests that USP37 may be involved in chemoresistance in osteosarcoma patients. Taken together, the immunohistochemistry study supports the findings from the in vitro and in silico experiments and suggests that USP37 and PCNA may play important roles in the progression of osteosarcoma.

## Discussion

Osteosarcoma is challenging cancer for oncologists because even after the surgical removal of tumors, 80% of patients develop lung metastasis [57]. The development of new drugs for osteosarcoma has been stagnant, with cisplatin remaining the primary adjuvant therapy for nearly 20 years. Combination chemotherapy after surgery has improved long-term survival rates by 60–70% for patients under 40 years old, but those with metastasis still have a poor prognosis with long-term survival rates of only 10–15% [58]. Despite efforts over the last 30 years, there has been no significant improvement in outcomes due to the difficulty in identifying pathogenomic mutations, particularly in the Rb1 and p53 pathways. [59]. MYC expression, a protein stabilized by USP37, is strongly related to many sarcomas, including osteosarcoma [24]. Understanding the oncogenic network of USP37 could lead to the development of new therapeutic modalities for osteosarcoma.

The ubiquitin proteasomal system controls protein stability by attaching or cleaving polyubiquitin chains, making it an attractive target for cancer therapeutics. The FDA has approved the proteasomal inhibitor Bortezomib for the treatment of mantle cell lymphoma and multiple myeloma [60]. USP37 is a Deubiquitinating enzyme that cleaves attached ubiquitin, destabilizing oncoproteins and regulating critical cellular processes in various cancer types[61, 22]. USP37 stabilizes MYC in lung cancer and the oncogenic fusion protein PLZF/RARA in Acute promyelocytic leukaemia (APL) [24, 20]. In Clear cell renal cell carcinoma (ccRCC), USP37 binds to and stabilizes HIF2α, promoting kidney cancer tumorigenesis [16]. Depletion of USP37 leads to increased sensitivity to agents that induce replication stress by destabilizing checkpoint kinase. We have provided evidence that the CHK 1 is destabilized in the absence of USP37, thereby effecting its function. The study found that in addition to CHK1, several other replication factors at the replication fork correlated with USP37. Our analysis of 44 cell lines indicated that CHK1 has the strongest correlation with USP37 in addition to other replication factors [23]. Cyclin A and CDT1, among others, have been shown to interact with USP37, controlling replication checkpoint and replication fork stability when exposed to replication stress [14, 18]. USPs have been found to be overexpressed in osteosarcoma cells, and inhibiting USPs with a Pan USP inhibitor led to the inhibition of osteosarcoma cell growth and reduced metastasis. TCGA data from 262 osteosarcoma patients indicated that USP37 transcript levels were significantly elevated and correlated with reduced OS and DFS in patients (Fig. 1A, B). In vitro analysis showed that USP37 transcript and protein levels were significantly elevated in osteosarcoma cell lines U20S and MG63 compared to non-transformed cell line MCF 10A (Fig. 1C, D).

The study conducted RNA sequencing analysis in osteosarcoma cells to better understand the transcriptome regulated by USP37 and the possible pathways it regulates. The analysis indicated a distinct pattern of gene expression when USP37 was overexpressed or depleted, identified dysregulated gene sets, and determined gene ontologies (Fig. 2C, D, E). The KEGG and reactome pathway analysis showed that USP37 overexpression activates several distinct pathways in osteosarcoma cells, including Interferon signaling, Acute phase response signaling, Production of ROS, and HIF 1 alpha signaling (Additional file 1: Figure S4A, B). Interestingly, not many replicative genes associated with USP37 were found in the analysis, but these genes need to be validated as they may provide insight into the physiological role of USP37 beyond cancer. Recent findings have shown that USP37 is the target gene of Topoisomerase IIA (TOP2a), an important nuclear enzyme that resolves entanglements and stimulates adult neurogenesis [29].

The study examined the role of USP37, a protein that regulates DNA replicative stress, in osteosarcoma cells. Elevated levels of USP37 were found in osteosarcoma cells, and it was discovered that overexpression of USP37 led to enhanced survival in response to replication stress, while its depletion led to reduced survival (Fig. 4A). Induction of replication stress led to an increase in USP37 expression in osteosarcoma cells, which correlated with elevated levels of the DNA damage marker, γH2AX (Fig. 4B, C, D). USP37 was shown to be highly expressed in the cytoplasm of breast cancer patients and is an independent poor prognostic parameter for overall survival, recurrence-free survival, and metastasis-free survival [30]. So we assessed the distribution of USP37 in osteosarcoma cells after replication stress and it was seen that on induction of replication stress and isolation of cytoplasmic and nuclear fraction there was an increase in cytoplasmic fraction of USP37 implying movement of USP37 from nucleus to cytoplasm (Fig. 4E). Depletion of USP37 led to the spontaneous accumulation of γH2AX foci, suggesting that USP37 may play a role in basal DNA damage response (Fig. 5A). When these cells were further treated with HU, there was an increase in the number of γH2AX foci as compared to control Si RNA (Fig. 5B). We further treated cells with cisplatin which induces both DNA single-strand and double-strand breaks and again showed an increase in the number of γH2AX foci (Fig. 5D). We also looked upon the accumulation of 53BP1 foci which is the preferential marker for measuring DNA double-strand break, and an increase in the number of 53BP1 foci was seen in USP37 depleted cells as compared to si Control in HU treated cells (Additional file 1: Figure S6B). In order to validate our finding further, we treated cells that overexpressed USP37 with HU and immuno-stained osteosarcoma cells with Anti USP37 antibody as well as Anti 53BP1 and Anti Replication protein A antibody to assess to resolution of these DNA damage foci in cells that were over expressing USP37 as compared to cells in which its expression was low. As indicated in (Fig. 5E.) cells with low expression of USP37 (Marked with blue arrow) have high number of unresolved RPA and 53BP1 foci as compared to cells that have a high level of USP37 (Marked with Orange arrow), indicating that USP37 over expressing cells were able to resolve the DNA damage defects easily as compared to cells that express low level of USP37.

In our recent study, we analyzed the available proteomic profile of different cell lines and found that USP37 showed variable correlation with different replication factors and DNA damage proteins, including Chk1 and PCNA, which is a key protein in replication fork and functions as a docking interface for different replication fork factor by working as a sliding clamp loader as DNA replication fork progresses [23, 50–52]. PCNA is a important protein in DNA replication machinery, which is required as a processivity factor for DNA polymerase and is one of the interacting partners in a loop of numerous other proteins involved in DNA replication and repair. Studies have shown that USP37 can interact with and deubiquitinate several proteins involved in the DNA damage response and thereby promote DNA repair. Moreover, USP37 has been shown to be important for the recruitment of DNA repair factors to sites of DNA damage and for the resolution of DNA repair intermediates. One of the study study suggested that CDT1 regulates DNA replication by regulating its phosphorylated form and regulating fork speed [18]. The results of this study were further supported by Li et al. [19]. PCNA emerged as a key candidate in the analysis of putative targets, so we investigated whether overexpression of USP37 stabilizes PCNA in response to replication stress.. We found that cells overexpressing USP37 showed enhanced time course stabilization of PCNA in a cyclohexamide chase experiment compared to cells in which USP37 was not overexpressed. (Fig. 6A, B). Endogenous USP37 also interacted with PCNA in both the absence and presence of replication stress, and this interaction was confirmed through both forward and reverse immunoprecipitation (Fig. 6C, D). We also overexpressed USP37 and performed immunocapture using Myc tag antibody to verify our results in both forward and reverse Immuno precipitation (Fig. 6E, F). We investigated whether USP37 is involved in the deubiquitination of PCNA, as it was shown to interact with PCNA. Previous studies have shown that PCNA interacts with USP1 to deubiquitinate it [53] while USP7 can modulate UV-induced PCNA monoubiquitination by regulating DNA polymerase eta stability [54]. We hypothesized that USP37 may be responsible for deubiquitinating PCNA. However, our experiments revealed that overexpression of either USP37 or its kinase mutant did not affect PCNA ubiquitination. (Fig. 6G, H). This suggests that PCNA may act as a scaffold for USP37 activity, aiding in the recruitment of other replication factors like CDT1, as reported previously. [18, 19]. We did observe deubiquitination of CHK1 in the same assay, consistent with earlier reports.

Given that we have previously demonstrated USP37's interaction with CHK1 its ability to enhance CHK1 activity [23], both of which are closely associated with replication fork movement, and that USP37 is also known to play a crucial role in the Homologous Recombination pathway [55], we conducted a DNA fiber assay to evaluate replication fork movement at the single molecule level. Our analysis revealed that USP37-depleted cells had much shorter DNA replicons, a decrease in the percentage of DNA fibers, and fewer forks, indicating compromised DNA synthesis as compared to USP37-proficient cells (Fig. 7B, D). Additionally, exposure of the cells to replication stress resulted in a further reduction in USP37-depleted cells and an increase in stalled replication forks. These results demonstrate that USP37 is critical for replication fork movement by interacting with PCNA and deubiquitinating replication factors like CDT1, as well as likely other factors, to facilitate DNA replication fork progression.

**Fig. 10:**
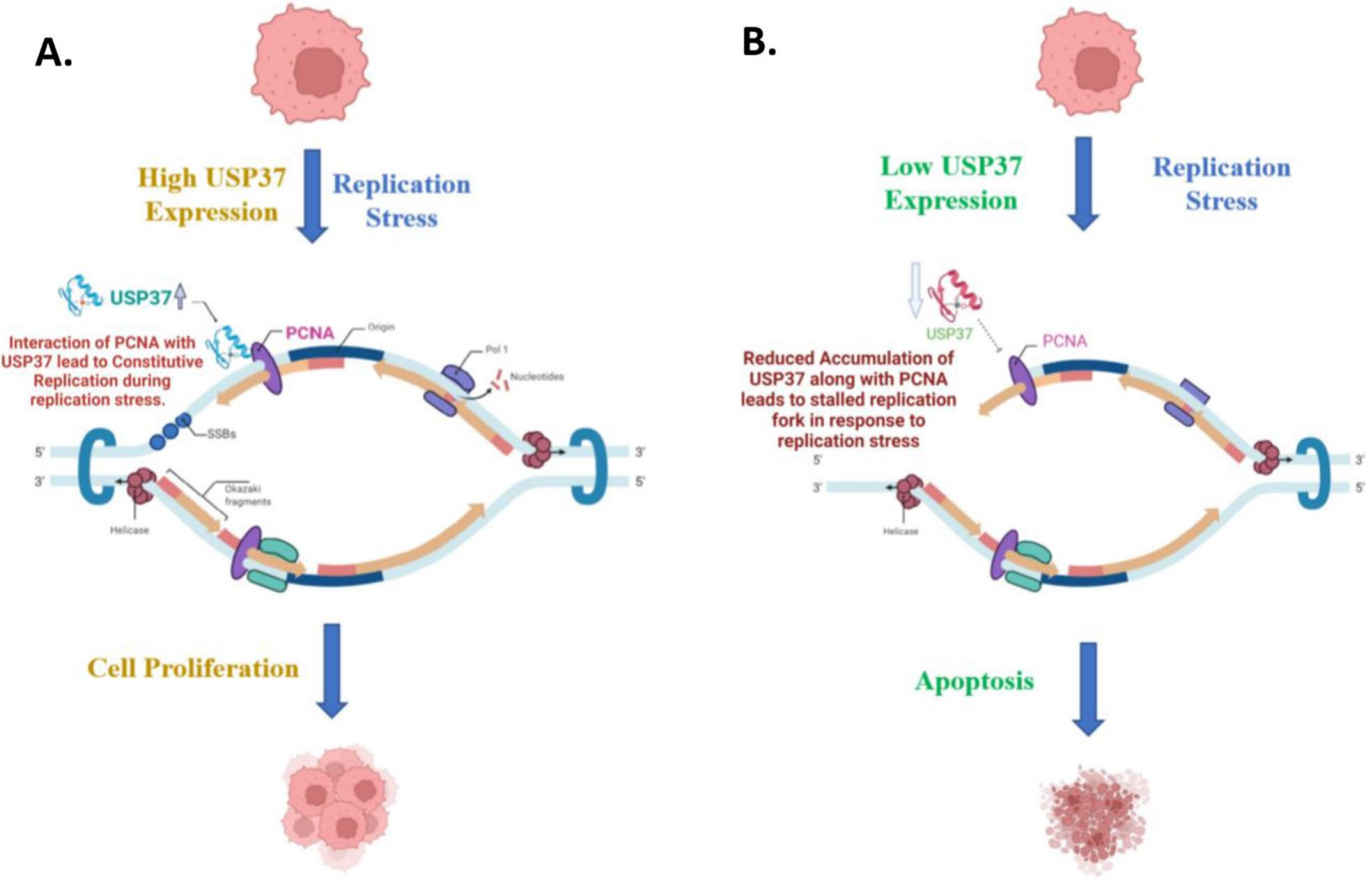
Graphical representation of Interaction of USP37 and PCNA. **A** Molecular mechanism of PCNA mediated interaction of USP37 with PCNA when USP37 levels are upregulated in osteosarcoma cells and molecular implications in the recruitment of additional replication proteins. **B** Molecular mechanism of PCNA mediated interaction of USP37 with PCNA when USP37 levels are downregulated in osteosarcoma cells and its molecular implications in the recruitment of additional replication proteins

In summary, our research findings demonstrate that the basic USP37 PH domain recognizes the acidic surface of PCNA and DNA minor groove at different residues (Fig. 8C, D and Table 1). This interaction between USP37 and PCNA plays a vital role in stabilizing different replication factors at the replication fork. Our in-silico analysis showed that the complex was stable throughout the 100 ns run, providing evidence that the PCNA DNA complex at the replication fork is the preferential binding site of USP37 (Fig. 8B). To test the clinical relevance of our findings, we analyzed the co-expression of USP37 and PCNA in a retrospective cohort of 40 osteosarcoma patients. Our analysis revealed that USP37 levels were significantly higher in osteosarcoma tissues as compared to non-transformed tissue and that the expression of USP37 had a positive correlation with PCNA expression (Fig. 9). Furthermore, elevated expression of USP37 was observed in patients who had undergone therapeutic intervention. Overall, our findings suggest that high expression or co-expression of USP37 and PCNA could be a predictor of therapeutic outcome in osteosarcoma patients. Recent studies have linked USP37 to replication stress and have shown its role in maintaining replication fork stability during genotoxic stress. These studies have identified CDT1 as one of the proteins deubiquitinated at the replication fork. [18, 19, 23, 55]. Our study has found that USP37 interacts with PCNA, a central replication factor, and has a higher affinity for PCNA-DNA complex than for PCNA alone.We propose a model where USP37 is recruited to the replication fork by interacting with PCNA at high or physiological levels of USP37. This causes the deubiquitination of additional replication factors such as CDT1, facilitating constitutive replication fork movement and supporting osteosarcoma cell proliferation (Fig. 10A). Depleting USP37 in osteosarcoma cells results in reduced recruitment to the replication fork, leading to instability of binding proteins, stalled replication fork, and enhanced apoptosis (Fig. 10B). Our study suggests that targeting USP37 can induce synthetic lethality in osteosarcoma by disrupting its oncogenic network and inducing replication stress.

## Conclusion

In this study, we have discovered that USP37, a protein of interest, interacts with PCNA, a critical protein involved in DNA replication. Our findings show that in osteosarcoma cells, USP37's interaction with PCNA plays a role in supporting the stability of the replication fork. We have also found that the expression of USP37 is linked to PCNA in archived osteosarcoma patient samples. Based on our observations, we propose that PCNA serves as a docking site for replication factors regulated by USP37, which helps the cells to manage replication stress associated with the tumor microenvironment.

## Supplementary Information

**Additional file 1: Figure S1.** Flowchart to depict workflow for Next Generation sequencing analysis. **Figure S2.** A. Expression analysis of USP37 in sarcoma through GEPIA2 matching normal TCGA and GTEx datasets showing transcripts per million of USP37 in tumor and normal sarcoma samples. B. Co-relation analysis of USP37 and PCNA in tumor and normal sarcoma samples though GEPIA2. C. Quantification of overexpression and Si RNA mediated depletion of USP37 in osteosarcoma cell line using Real-time PCR Assay (Relative Units RU). D. USP37 Overexpression and depletion in osteosarcoma cell lines. Cells were lysed and protein was resolved using SDS PAGE and probed using anti USP37 antibody. **Figure S3.** A. DEGs profiling of U2OS after USP37 overexpression. The heat map shows the statistically significant dysregulated genes/RNAs (± 2 Log2) of U2OS cells via whole RNA sequencing after USP37 overexpression with respect to normal untreated cells. B.DEGs profiling of U2OS after USP37 KO. The heat map shows the statistically significant dysregulated genes/RNAs (± 2 Log2) of U2OS cells via whole RNA sequencing after USP37 KO with respect to normal untreated cells. **Figure S4.** A. KEGG and Reactome pathways of downregulated and upregulated genes after USP37 overexpression in U2OS cells by cluego. B. KEGG and Reactome pathways of downregulated and upregulated genes after USP37 KO in U2OS cells by cluego. **Figure S5.** String Network of Upregulated and downregulated genes after USP37 Overexpression (A) and USP37 KO (B) with confidence level 0.4. **Figure S6.** USP37 interaction map by Biogrid. i Interactions with High throughput screening ii. Interactions without High throughput screening. **Figure S7.** A. U2OS osteosarcoma control cells and cells depleted of USP37 were treated with HU for 24 h and stained with Anti γH2Ax antibody to assess DNA damage response. B. U2OS osteosarcoma control cells and cells depleted of USP37 were treated with HU for 24 h and stained with Anti 53BP1 antibody to assess D NA damage response. **Figure S8.** MD simulation parameter of (A) USP37 full length and (B) USP37-PCNA-DNA complex. The figure represents SASA, Rg, Complex RMSD and RMSF for both USP37 full length and USP37-PCNA-DNA complex after 100ns of MD simulations.

**Additional file 2: Table S1.** Reagent and Resources.

**Additional file 3: Table S2.** List of differentially expressed genes between WT VS OE.

**Additional file 4: Table S3.** List of differentially expressed genes between WT VS KO.

**Additional file 5: Table S4.** List of differentially expressed genes between OE VS KO.

**Additional file 6: Table S5.** Osteosarcoma Patient related information.

## Data Availability

None.

## Abbreviations

TCGA: The cancer genome atlas
DUBs: Deubiquitinating enzymes
USP37: Ubiquitin specific protease 37
TPM: Transcripts per million
OS: Overall survival
DFS: Disease free survival
WT: Wild type
OE: Overexpression
KO: Knock down
DEGs: Differential expressed genes
HU: Hydroxyurea
CP: Cisplatin
PCNA: Proliferating cell nuclear antigen
CHX: Cycloheximide
HR: Homologous recombination
CIdU: 5-Chlorodeoxyuridine
IdU: 5-Iododeoxyuridine
UIM: Ubiquitin interacting motifs
TOP2a: Topoisomerase IIA
