# Supplementary material for: Ubiquitin specific peptidase 37 and PCNA interaction promotes osteosarcoma pathogenesis by modulating replication fork progression
Source: J Transl Med. 2023 Apr 28;21:286. doi: 10.1186/s12967-023-04126-2 (PMC10142227; doi:10.1186/s12967-023-04126-2)
Supplement: Supplementary file 1 — Additional file 1: Figure S1. Flowchart to depict workflow for Next Generation sequencing analysis. Figure S2. A. Expression analysis of USP37 in sarcoma through GEPIA2 matching normal TCGA and GTEx datasets showing transcripts per million of USP37 in tumor and normal sarcoma samples. B. Co-relation analysis of USP37 and PCNA in tumor and normal sarcoma samples though GEPIA2. C. Quantification of overexpression and Si RNA mediated depletion of USP37 in osteosarcoma cell line using Real-time PCR Assay (Relative Units RU). D. USP37 Overexpression and depletion in osteosarcoma cell lines. Cells were lysed and protein was resolved using SDS PAGE and probed using anti USP37 antibody. Figure S3. A. DEGs profiling of U2OS after USP37 overexpression. The heat map shows the statistically significant dysregulated genes/RNAs (± 2 Log2) of U2OS cells via whole RNA sequencing after USP37 overexpression with respect to normal untreated cells. B.DEGs profiling of U2OS after USP37 KO. The heat map shows the statistically significant dysregulated genes/RNAs (± 2 Log2) of U2OS cells via whole RNA sequencing after USP37 KO with respect to normal untreated cells. Figure S4. A. KEGG and Reactome pathways of downregulated and upregulated genes after USP37 overexpression in U2OS cells by cluego. B. KEGG and Reactome pathways of downregulated and upregulated genes after USP37 KO in U2OS cells by cluego. Figure S5. String Network of Upregulated and downregulated genes after USP37 Overexpression (A) and USP37 KO (B) with confidence level 0.4. Figure S6. USP37 interaction map by Biogrid. i Interactions with High throughput screening ii. Interactions without High throughput screening. Figure S7. A. U2OS osteosarcoma control cells and cells depleted of USP37 were treated with HU for 24 h and stained with Anti γH2Ax antibody to assess DNA damage response. B. U2OS osteosarcoma control cells and cells depleted of USP37 were treated with HU for 24 h and stained with Anti 53BP1 antibody to assess D [file 12967_2023_4126_MOESM1_ESM.pptx]

## Slide 1
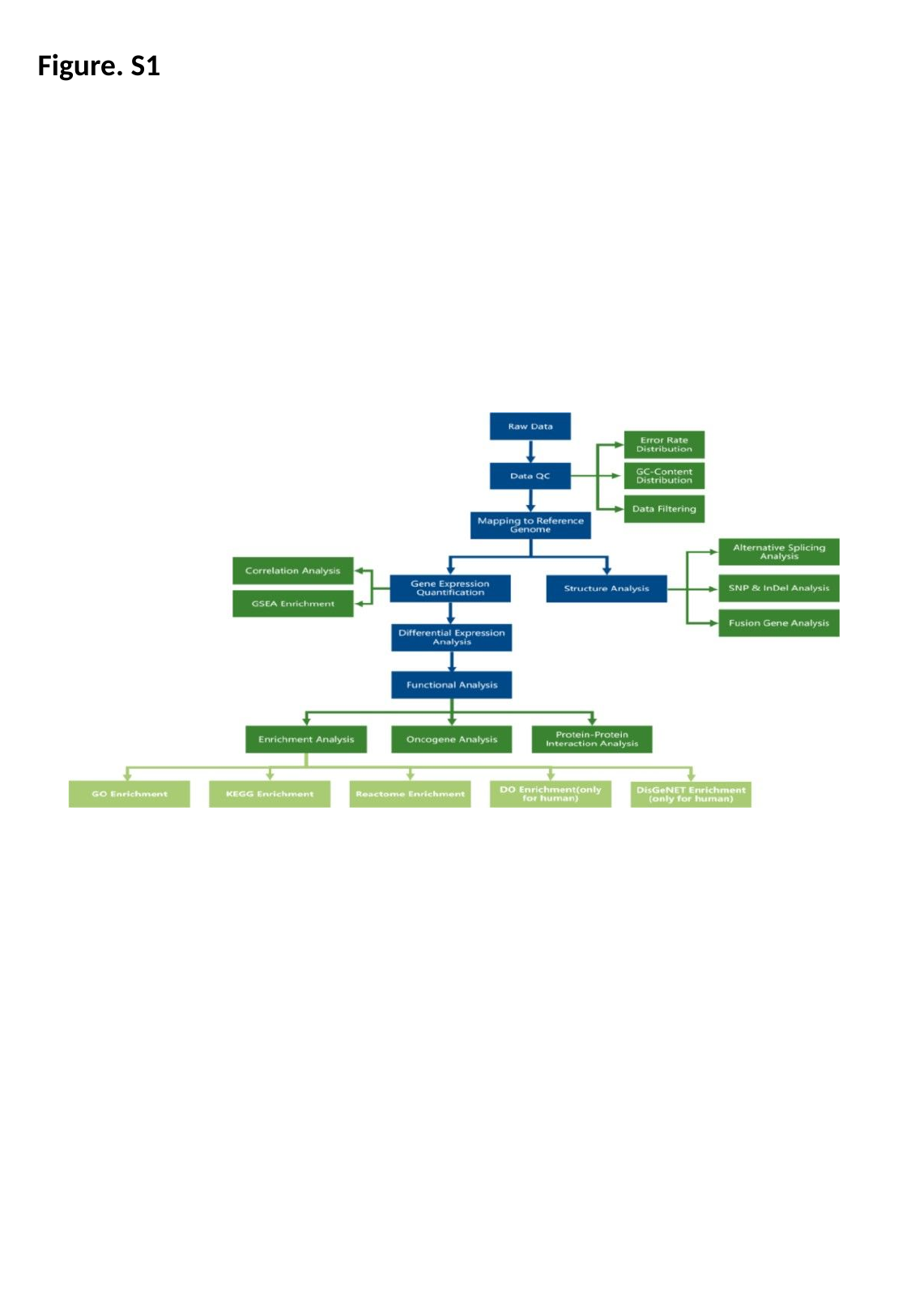

Figure. S1

## Slide 2
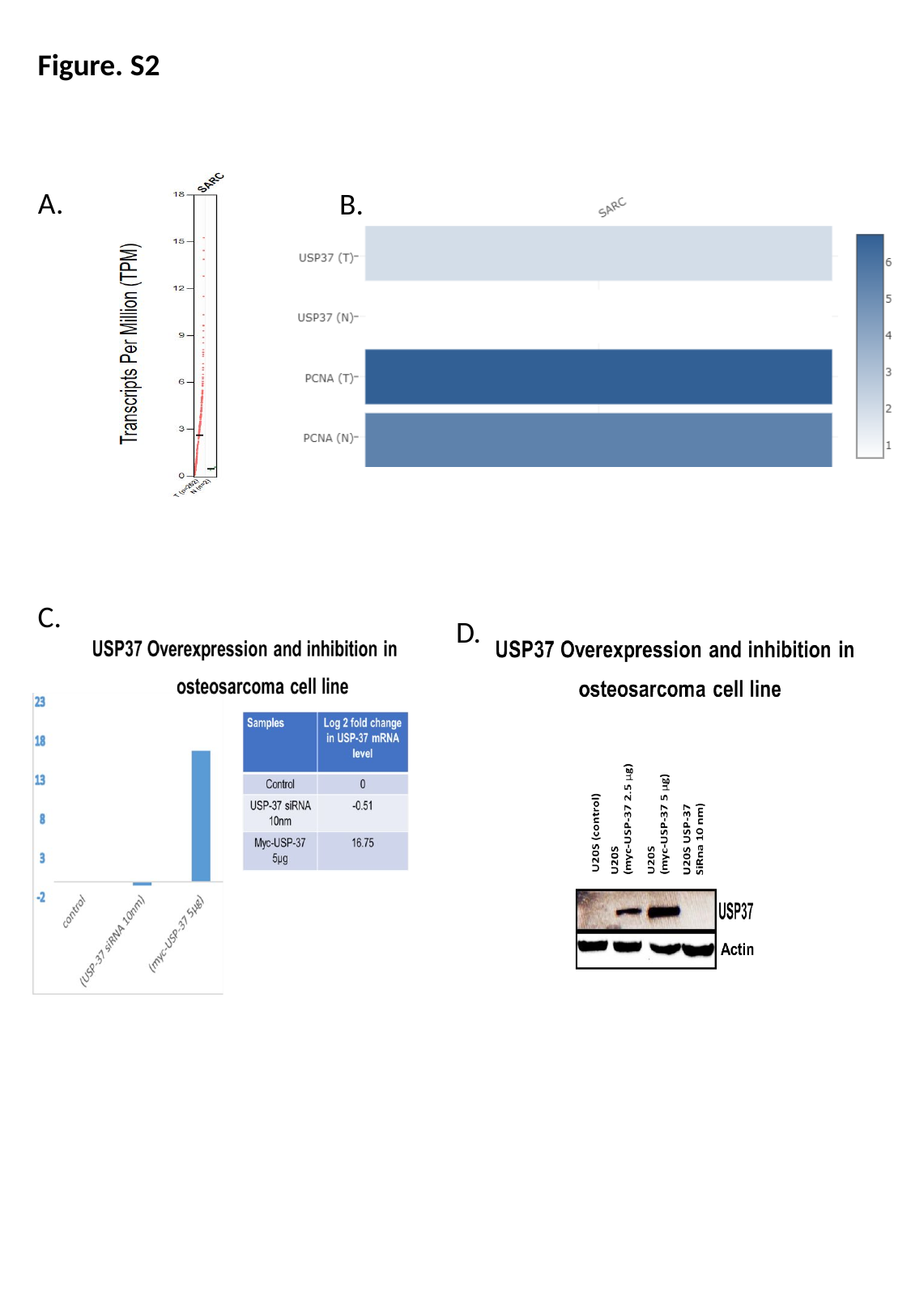

Figure. S2
A.
B.
C.
D.

## Slide 3
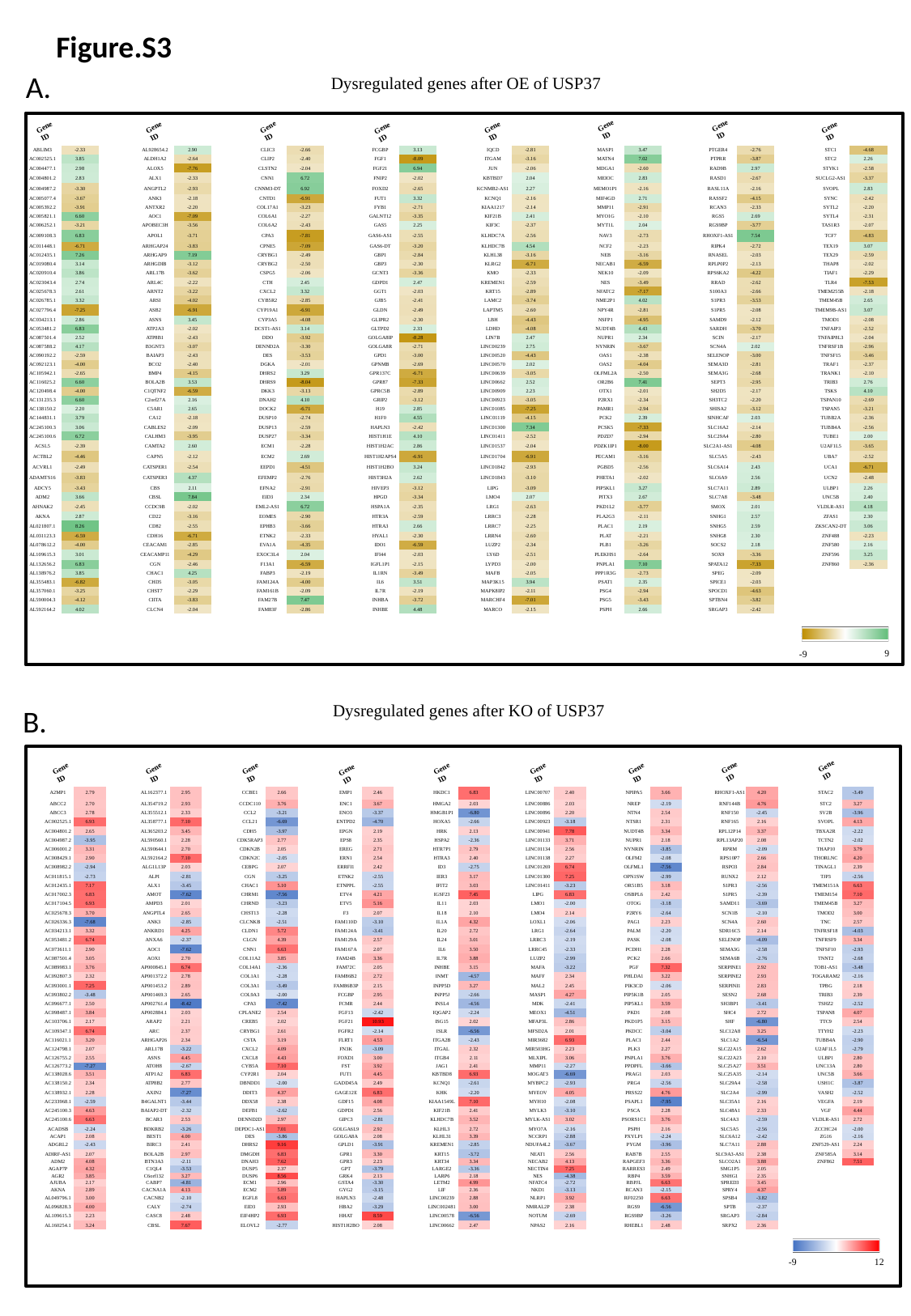

Figure.S3
A.
Dysregulated genes after OE of USP37
Gene ID
Gene ID
Gene ID
Gene ID
Gene ID
Gene ID
Gene ID
Gene ID
| ABLIM3 | -2.33 | | AL928654.2 | 2.90 | | CLIC3 | -2.66 | | FCGBP | 3.13 | | IQCD | -2.81 | | MASP1 | 3.47 | | PTGER4 | -2.76 | | STC1 | -4.68 |
| --- | --- | --- | --- | --- | --- | --- | --- | --- | --- | --- | --- | --- | --- | --- | --- | --- | --- | --- | --- | --- | --- | --- |
| AC002525.1 | 3.85 | | ALDH1A2 | -2.64 | | CLIP2 | -2.40 | | FGF1 | -8.09 | | ITGAM | -3.16 | | MATN4 | 7.02 | | PTPRR | -3.87 | | STC2 | 2.26 |
| AC004477.1 | 2.98 | | ALOX5 | -7.76 | | CLSTN2 | -2.04 | | FGF21 | 6.94 | | JUN | -2.06 | | MDGA1 | -2.60 | | RAD9B | 2.97 | | STYK1 | -2.58 |
| AC004801.2 | 2.83 | | ALX1 | -2.33 | | CNN1 | 6.72 | | FNIP2 | -2.02 | | KBTBD7 | 2.04 | | MEIOC | 2.83 | | RASD1 | -2.67 | | SUCLG2-AS1 | -3.37 |
| AC004987.2 | -3.30 | | ANGPTL2 | -2.93 | | CNNM3-DT | 6.92 | | FOXD2 | -2.65 | | KCNMB2-AS1 | 2.27 | | MEMO1P1 | -2.16 | | RASL11A | -2.16 | | SVOPL | 2.83 |
| AC005077.4 | -3.67 | | ANK3 | -2.18 | | CNTD1 | -6.91 | | FUT1 | 3.32 | | KCNQ1 | -2.16 | | MIF4GD | 2.71 | | RASSF2 | -4.15 | | SYNC | -2.42 |
| AC005392.2 | -3.91 | | ANTXR2 | -2.20 | | COL17A1 | -3.23 | | FYB1 | -2.71 | | KIAA1217 | -2.14 | | MMP11 | -2.91 | | RCAN3 | -2.33 | | SYTL2 | -2.20 |
| AC005821.1 | 6.60 | | AOC1 | -7.09 | | COL6A1 | -2.27 | | GALNT12 | -3.35 | | KIF21B | 2.41 | | MYO1G | -2.10 | | RGS5 | 2.69 | | SYTL4 | -2.31 |
| AC006252.1 | -3.21 | | APOBEC3H | -3.56 | | COL6A2 | -2.43 | | GAS5 | 2.25 | | KIF3C | -2.37 | | MYT1L | 2.04 | | RGS9BP | -3.77 | | TAS1R3 | -2.07 |
| AC009108.3 | 6.83 | | APOL1 | -3.71 | | CPA3 | -7.81 | | GAS6-AS1 | -2.55 | | KLHDC7A | -2.56 | | NAV3 | -2.73 | | RHOXF1-AS1 | 7.54 | | TCF7 | -4.83 |
| AC011448.1 | -6.71 | | ARHGAP24 | -3.83 | | CPNE5 | -7.09 | | GAS6-DT | -3.20 | | KLHDC7B | 4.54 | | NCF2 | -2.23 | | RIPK4 | -2.72 | | TEX19 | 3.07 |
| AC012435.1 | 7.26 | | ARHGAP9 | 7.19 | | CRYBG1 | -2.49 | | GBP1 | -2.84 | | KLHL38 | -3.16 | | NEB | -3.16 | | RNASEL | -2.03 | | TEX29 | -2.59 |
| AC019080.4 | 3.14 | | ARHGDIB | -3.12 | | CRYBG2 | -2.50 | | GBP3 | -2.30 | | KLRG2 | -6.71 | | NECAB1 | -6.59 | | RPLP0P2 | -2.13 | | THAP8 | -2.02 |
| AC020910.4 | 3.86 | | ARL17B | -3.62 | | CSPG5 | -2.06 | | GCNT3 | -3.36 | | KMO | -2.33 | | NEK10 | -2.09 | | RPS6KA2 | -4.22 | | TIAF1 | -2.29 |
| AC023043.4 | 2.74 | | ARL4C | -2.22 | | CTH | 2.45 | | GDPD1 | 2.47 | | KREMEN1 | -2.59 | | NES | -3.49 | | RRAD | -2.62 | | TLR4 | -7.53 |
| AC025678.3 | 2.61 | | ARNT2 | -3.22 | | CXCL2 | 3.32 | | GGT1 | -2.03 | | KRT15 | -2.89 | | NFATC2 | -7.17 | | S100A3 | -2.66 | | TMEM255B | -2.18 |
| AC026785.1 | 3.32 | | ARSI | -4.02 | | CYB5R2 | -2.85 | | GJB5 | -2.41 | | LAMC2 | -3.74 | | NME2P1 | 4.02 | | S1PR3 | -3.53 | | TMEM45B | 2.65 |
| AC027796.4 | -7.25 | | ASB2 | -6.91 | | CYP19A1 | -6.91 | | GLDN | -2.49 | | LAPTM5 | -2.60 | | NPY4R | -2.81 | | S1PR5 | -2.08 | | TMEM9B-AS1 | 3.07 |
| AC034213.1 | 2.86 | | ASNS | 3.45 | | CYP3A5 | -4.08 | | GLIPR2 | -2.30 | | LBH | -4.43 | | NSFP1 | -4.95 | | SAMD9 | -2.12 | | TMOD1 | -2.08 |
| AC053481.2 | 6.83 | | ATP2A3 | -2.02 | | DCST1-AS1 | 3.14 | | GLTPD2 | 2.33 | | LDHD | -4.08 | | NUDT4B | 4.43 | | SARDH | -3.70 | | TNFAIP3 | -2.52 |
| AC087501.4 | 2.52 | | ATP8B1 | -2.43 | | DDO | -3.92 | | GOLGA8IP | -8.28 | | LIN7B | 2.47 | | NUPR1 | 2.34 | | SCIN | -2.17 | | TNFAIP8L3 | -2.04 |
| AC087588.2 | 4.17 | | B3GNT3 | -3.07 | | DENND2A | -3.30 | | GOLGA8R | -2.71 | | LINC00239 | 2.75 | | NYNRIN | -3.67 | | SCN4A | 2.02 | | TNFRSF1B | -2.96 |
| AC090192.2 | -2.59 | | BAIAP3 | -2.43 | | DES | -3.53 | | GPD1 | -3.00 | | LINC00520 | -4.43 | | OAS1 | -2.38 | | SELENOP | -3.00 | | TNFSF15 | -3.46 |
| AC092123.1 | -4.00 | | BCO2 | -2.40 | | DGKA | -2.01 | | GPNMB | -2.69 | | LINC00570 | 2.02 | | OAS2 | -4.04 | | SEMA3D | -2.81 | | TRAF1 | -2.37 |
| AC105942.1 | -2.65 | | BMP4 | -4.15 | | DHRS2 | 3.29 | | GPR137C | -6.71 | | LINC00639 | -3.05 | | OLFML2A | -2.50 | | SEMA3G | -2.68 | | TRANK1 | -2.10 |
| AC116025.2 | 6.60 | | BOLA2B | 3.53 | | DHRS9 | -8.04 | | GPR87 | -7.33 | | LINC00662 | 2.52 | | OR2B6 | 7.41 | | SEPT3 | -2.95 | | TRIB3 | 2.76 |
| AC120498.4 | -4.00 | | C1QTNF2 | -6.59 | | DKK3 | -3.13 | | GPRC5B | -2.89 | | LINC00909 | 2.23 | | OTX1 | -2.01 | | SH2D5 | -2.17 | | TSKS | 4.10 |
| AC131235.3 | 6.60 | | C2orf27A | 2.16 | | DNAH2 | 4.10 | | GRIP2 | -3.12 | | LINC00923 | -3.05 | | P2RX1 | -2.34 | | SH3TC2 | -2.20 | | TSPAN10 | -2.69 |
| AC138150.2 | 2.20 | | C5AR1 | 2.65 | | DOCK2 | -6.71 | | H19 | 2.85 | | LINC01085 | -7.25 | | PAMR1 | -2.94 | | SHISA2 | -3.12 | | TSPAN5 | -3.21 |
| AC144831.1 | 3.79 | | CA12 | -2.18 | | DUSP10 | -2.74 | | H1F0 | 4.55 | | LINC01119 | -4.15 | | PCK2 | 2.39 | | SINHCAF | 2.03 | | TUBB2A | -2.36 |
| AC245100.3 | 3.06 | | CABLES2 | -2.09 | | DUSP13 | -2.59 | | HAPLN3 | -2.42 | | LINC01300 | 7.34 | | PCSK5 | -7.33 | | SLC16A2 | -2.14 | | TUBB4A | -2.56 |
| AC245100.6 | 6.72 | | CALHM3 | -3.95 | | DUSP27 | -3.34 | | HIST1H1E | 4.10 | | LINC01411 | -2.52 | | PDZD7 | -2.94 | | SLC29A4 | -2.80 | | TUBE1 | 2.00 |
| ACSL5 | -2.39 | | CAMTA2 | 2.60 | | ECM1 | -2.28 | | HIST1H2AC | 2.86 | | LINC01537 | -2.04 | | PDZK1IP1 | -8.00 | | SLC2A1-AS1 | -4.08 | | U2AF1L5 | -3.65 |
| ACTBL2 | -4.46 | | CAPN5 | -2.12 | | ECM2 | 2.69 | | HIST1H2APS4 | -6.91 | | LINC01704 | -6.91 | | PECAM1 | -3.16 | | SLC5A5 | -2.43 | | UBA7 | -2.52 |
| ACVRL1 | -2.49 | | CATSPER1 | -2.54 | | EEPD1 | -4.51 | | HIST1H2BO | 3.24 | | LINC01842 | -2.93 | | PGBD5 | -2.56 | | SLC6A14 | 2.43 | | UCA1 | -6.71 |
| ADAMTS16 | -3.83 | | CATSPER3 | 4.37 | | EFEMP2 | -2.76 | | HIST3H2A | 2.62 | | LINC01843 | -3.10 | | PHETA1 | -2.02 | | SLC6A9 | 2.56 | | UCN2 | -2.48 |
| ADCY5 | -3.43 | | CBS | 2.11 | | EFNA2 | -2.91 | | HIVEP3 | -3.12 | | LIPG | -3.09 | | PIP5KL1 | 3.27 | | SLC7A11 | 2.89 | | ULBP1 | 2.26 |
| ADM2 | 3.66 | | CBSL | 7.84 | | EID3 | 2.34 | | HPGD | -3.34 | | LMO4 | 2.07 | | PITX3 | 2.67 | | SLC7A8 | -3.48 | | UNC5B | 2.40 |
| AHNAK2 | -2.45 | | CCDC9B | -2.02 | | EML2-AS1 | 6.72 | | HSPA1A | -2.35 | | LRG1 | -2.63 | | PKD1L2 | -3.77 | | SMOX | 2.01 | | VLDLR-AS1 | 4.18 |
| AKNA | 2.87 | | CD22 | -3.16 | | EOMES | -2.90 | | HTR3A | -2.59 | | LRRC3 | -2.28 | | PLA2G3 | -2.11 | | SNHG1 | 2.57 | | ZFAS1 | 2.30 |
| AL021807.1 | 8.26 | | CD82 | -2.55 | | EPHB3 | -3.66 | | HTRA3 | 2.66 | | LRRC7 | -2.25 | | PLAC1 | 2.19 | | SNHG5 | 2.59 | | ZKSCAN2-DT | 3.06 |
| AL031123.3 | -6.59 | | CDH16 | -6.71 | | ETNK2 | -2.33 | | HYAL1 | -2.30 | | LRRN4 | -2.60 | | PLAT | -2.21 | | SNHG8 | 2.30 | | ZNF488 | -2.23 |
| AL078612.2 | -4.00 | | CEACAM1 | -2.85 | | EVA1A | -4.35 | | IDO1 | -6.59 | | LUZP2 | -2.34 | | PLB1 | -3.26 | | SOCS2 | 2.18 | | ZNF580 | 2.16 |
| AL109615.3 | 3.01 | | CEACAMP11 | -4.29 | | EXOC3L4 | 2.04 | | IFI44 | -2.03 | | LY6D | -2.51 | | PLEKHS1 | -2.64 | | SOX9 | -3.36 | | ZNF596 | 3.25 |
| AL132656.2 | 6.83 | | CGN | -2.46 | | F13A1 | -6.59 | | IGFL1P1 | -2.15 | | LYPD3 | -2.00 | | PNPLA1 | 7.10 | | SPATA12 | -7.33 | | ZNF860 | -2.36 |
| AL138976.2 | 3.85 | | CHAC1 | 4.25 | | FABP3 | -2.19 | | IL1RN | -3.49 | | MAFB | -2.05 | | PPP1R3G | -2.73 | | SPEG | -2.09 | | | |
| AL355483.1 | -6.82 | | CHD5 | -3.05 | | FAM124A | -4.00 | | IL6 | 3.51 | | MAP3K15 | 3.94 | | PSAT1 | 2.35 | | SPICE1 | -2.03 | | | |
| AL357060.1 | -3.25 | | CHST7 | -2.29 | | FAM161B | -2.09 | | IL7R | -2.19 | | MAPK8IP2 | -2.11 | | PSG4 | -2.94 | | SPOCD1 | -4.63 | | | |
| AL590004.3 | -4.12 | | CIITA | -3.83 | | FAM27B | 7.47 | | INHBA | -3.72 | | MARCHF4 | -7.01 | | PSG5 | -3.43 | | SPTBN4 | -3.82 | | | |
| AL592164.2 | 4.02 | | CLCN4 | -2.04 | | FAM83F | -2.86 | | INHBE | 4.48 | | MARCO | -2.15 | | PSPH | 2.66 | | SRGAP3 | -2.42 | | | |
9
-9
Dysregulated genes after KO of USP37
B.
Gene ID
Gene ID
Gene ID
Gene ID
Gene ID
Gene ID
Gene ID
Gene ID
Gene ID
| A2MP1 | 2.79 | | AL162377.1 | 2.95 | | CCBE1 | 2.66 | | EMP1 | 2.46 | | HKDC1 | 6.83 | | LINC00707 | 2.40 | | NPIPA5 | 3.66 | | RHOXF1-AS1 | 4.20 | | STAC2 | -3.49 |
| --- | --- | --- | --- | --- | --- | --- | --- | --- | --- | --- | --- | --- | --- | --- | --- | --- | --- | --- | --- | --- | --- | --- | --- | --- | --- |
| ABCC2 | 2.70 | | AL354719.2 | 2.93 | | CCDC110 | 3.76 | | ENC1 | 3.67 | | HMGA2 | 2.03 | | LINC00886 | 2.03 | | NREP | -2.19 | | RNF144B | 4.76 | | STC2 | 3.27 |
| ABCC3 | 2.78 | | AL355512.1 | 2.33 | | CCL2 | -3.21 | | ENO3 | -3.37 | | HMGB1P1 | -6.80 | | LINC00896 | 2.20 | | NTN4 | 2.54 | | RNF150 | -2.45 | | SV2B | -3.96 |
| AC002525.1 | 6.93 | | AL358777.1 | 7.10 | | CCL21 | -6.69 | | ENTPD2 | -4.70 | | HOXA5 | -2.66 | | LINC00923 | -3.18 | | NTSR1 | 2.31 | | RNF165 | 2.16 | | SVOPL | 4.13 |
| AC004801.2 | 2.65 | | AL365203.2 | 3.45 | | CDH5 | -3.97 | | EPGN | 2.19 | | HRK | 2.13 | | LINC00941 | 7.78 | | NUDT4B | 3.34 | | RPL12P14 | 3.37 | | TBXA2R | -2.22 |
| AC004987.2 | -3.95 | | AL590560.1 | 2.28 | | CDK5RAP3 | 2.77 | | EPS8 | 2.35 | | HSPA2 | -2.36 | | LINC01133 | 3.71 | | NUPR1 | 2.18 | | RPL13AP20 | 2.08 | | TCTN2 | -2.02 |
| AC006001.2 | 3.31 | | AL590644.1 | 2.70 | | CDKN2B | 2.05 | | EREG | 2.71 | | HTR7P1 | 2.79 | | LINC01134 | 2.56 | | NYNRIN | -3.85 | | RPRM | -2.09 | | THAP10 | 3.79 |
| AC008429.1 | 2.90 | | AL592164.2 | 7.10 | | CDKN2C | -2.05 | | ERN1 | 2.54 | | HTRA3 | 2.40 | | LINC01138 | 2.27 | | OLFM2 | -2.08 | | RPS10P7 | 2.66 | | THORLNC | 4.20 |
| AC008982.2 | -2.94 | | ALG1L13P | 2.03 | | CEBPG | 2.07 | | ERRFI1 | 2.42 | | ID3 | -2.75 | | LINC01269 | 6.74 | | OLFML1 | -7.56 | | RSPO3 | 2.84 | | TINAGL1 | 2.39 |
| AC011815.1 | -2.73 | | ALPI | -2.81 | | CGN | -3.25 | | ETNK2 | -2.55 | | IER3 | 3.17 | | LINC01300 | 7.25 | | OPN1SW | -2.99 | | RUNX2 | 2.12 | | TJP3 | -2.56 |
| AC012435.1 | 7.17 | | ALX1 | -3.45 | | CHAC1 | 5.10 | | ETNPPL | -2.55 | | IFIT2 | 3.03 | | LINC01411 | -3.23 | | OR51B5 | 3.18 | | S1PR3 | -2.56 | | TMEM151A | 6.63 |
| AC017002.3 | 6.83 | | AMOT | -7.62 | | CHRM1 | -7.56 | | ETV4 | 4.21 | | IGSF23 | 7.45 | | LIPG | 6.83 | | OSBPL6 | 2.42 | | S1PR5 | -2.39 | | TMEM154 | 7.10 |
| AC017104.5 | 6.93 | | AMPD3 | 2.01 | | CHRND | -3.23 | | ETV5 | 5.16 | | IL11 | 2.03 | | LMO1 | -2.00 | | OTOG | -3.18 | | SAMD11 | -3.69 | | TMEM45B | 3.27 |
| AC025678.3 | 3.70 | | ANGPTL4 | 2.65 | | CHST13 | -2.28 | | F3 | 2.07 | | IL18 | 2.10 | | LMO4 | 2.14 | | P2RY6 | -2.64 | | SCN1B | -2.10 | | TMOD2 | 3.00 |
| AC026336.3 | -7.68 | | ANK3 | -2.85 | | CLCNKB | -2.51 | | FAM110D | -3.10 | | IL1A | 4.32 | | LOXL1 | -2.06 | | PAG1 | 2.23 | | SCN4A | 2.60 | | TNC | 2.57 |
| AC034213.1 | 3.32 | | ANKRD1 | 4.25 | | CLDN1 | 5.72 | | FAM124A | -3.41 | | IL20 | 2.72 | | LRG1 | -2.64 | | PALM | -2.20 | | SDR16C5 | 2.14 | | TNFRSF18 | -4.03 |
| AC053481.2 | 6.74 | | ANXA6 | -2.37 | | CLGN | 4.39 | | FAM129A | 2.57 | | IL24 | 3.01 | | LRRC3 | -2.19 | | PASK | -2.08 | | SELENOP | -4.09 | | TNFRSF9 | 3.34 |
| AC073611.1 | 2.90 | | AOC1 | -7.62 | | CNN1 | 6.63 | | FAM167A | 2.07 | | IL6 | 3.50 | | LRRC45 | -2.33 | | PCDH1 | 2.28 | | SEMA3G | -2.58 | | TNFSF10 | -2.93 |
| AC087501.4 | 3.05 | | AOX1 | 2.70 | | COL11A2 | 3.85 | | FAM24B | 3.36 | | IL7R | 3.88 | | LUZP2 | -2.99 | | PCK2 | 2.66 | | SEMA6B | -2.76 | | TNNT2 | -2.68 |
| AC089983.1 | 3.76 | | AP000845.1 | 6.74 | | COL14A1 | -2.36 | | FAM72C | 2.05 | | INHBE | 3.15 | | MAFA | -3.22 | | PGF | 7.32 | | SERPINE1 | 2.92 | | TOB1-AS1 | -3.48 |
| AC092807.3 | 2.32 | | AP001372.2 | 2.78 | | COL1A1 | -2.28 | | FAM86B2 | 2.72 | | INMT | -4.57 | | MAFF | 2.34 | | PHLDA1 | 3.22 | | SERPINE2 | 2.93 | | TOGARAM2 | -2.16 |
| AC093001.1 | 7.25 | | AP001453.2 | 2.89 | | COL3A1 | -3.49 | | FAM86B3P | 2.15 | | INPP5D | 3.27 | | MAL2 | 2.45 | | PIK3CD | -2.06 | | SERPINI1 | 2.83 | | TPBG | 2.18 |
| AC093802.2 | -3.48 | | AP001469.3 | 2.65 | | COL9A3 | -2.00 | | FCGBP | 2.95 | | INPP5J | -2.66 | | MASP1 | 4.27 | | PIP5K1B | 2.05 | | SESN2 | 2.68 | | TRIB3 | 2.39 |
| AC096677.1 | 2.50 | | AP002761.4 | -8.42 | | CPA3 | -7.42 | | FCMR | 2.44 | | INSL4 | -4.56 | | MDK | -2.41 | | PIP5KL1 | 3.59 | | SH3BP1 | -3.41 | | TSHZ2 | -2.52 |
| AC098487.1 | 3.84 | | AP002884.1 | 2.03 | | CPLANE2 | 2.54 | | FGF13 | -2.42 | | IQGAP2 | -2.24 | | MEOX1 | -4.51 | | PKD1 | 2.08 | | SHC4 | 2.72 | | TSPAN8 | 4.07 |
| AC103706.1 | 2.17 | | ARAP2 | 2.21 | | CREB5 | 2.02 | | FGF21 | 10.93 | | ISG15 | 2.02 | | MFAP3L | 2.86 | | PKD1P5 | 3.15 | | SHF | -6.80 | | TTC9 | 2.54 |
| AC109347.1 | 6.74 | | ARC | 2.37 | | CRYBG1 | 2.61 | | FGFR2 | -2.14 | | ISLR | -6.56 | | MFSD2A | 2.01 | | PKDCC | -3.04 | | SLC12A8 | 3.25 | | TTYH2 | -2.23 |
| AC116021.1 | 3.20 | | ARHGAP26 | 2.34 | | CSTA | 3.19 | | FLRT1 | 4.53 | | ITGA2B | -2.43 | | MIR3682 | 6.93 | | PLAC1 | 2.44 | | SLC1A2 | -6.54 | | TUBB4A | -2.90 |
| AC124798.1 | 2.07 | | ARL17B | -3.22 | | CXCL2 | 4.09 | | FN3K | -3.09 | | ITGAL | 2.32 | | MIR503HG | 2.23 | | PLK3 | 2.27 | | SLC22A15 | 2.62 | | U2AF1L5 | -2.79 |
| AC126755.2 | 2.55 | | ASNS | 4.45 | | CXCL8 | 4.43 | | FOXD1 | 3.00 | | ITGB4 | 2.11 | | MLXIPL | 3.06 | | PNPLA1 | 3.76 | | SLC22A23 | 2.10 | | ULBP1 | 2.80 |
| AC126773.2 | -7.27 | | ATOH8 | -2.67 | | CYB5A | 7.10 | | FST | 3.92 | | JAG1 | 2.41 | | MMP11 | -2.27 | | PPDPFL | -3.66 | | SLC25A27 | 3.51 | | UNC13A | 2.80 |
| AC138028.6 | 3.51 | | ATP1A2 | 6.83 | | CYP2R1 | 2.04 | | FUT1 | 4.45 | | KBTBD8 | 6.93 | | MOGAT3 | -6.69 | | PRAG1 | 2.03 | | SLC25A35 | -2.14 | | UNC5B | 3.66 |
| AC138150.2 | 2.34 | | ATP8B2 | 2.77 | | DBNDD1 | -2.00 | | GADD45A | 2.49 | | KCNQ1 | -2.61 | | MYBPC2 | -2.93 | | PRG4 | -2.56 | | SLC29A4 | -2.58 | | USH1C | -3.87 |
| AC138932.1 | 2.28 | | AXIN2 | -7.27 | | DDIT3 | 4.37 | | GAGE12E | 6.83 | | KHK | -2.20 | | MYEOV | 4.05 | | PRSS22 | 4.76 | | SLC2A4 | -2.99 | | VASH2 | -2.52 |
| AC233968.1 | -2.59 | | B4GALNT1 | -3.44 | | DDX58 | 2.38 | | GDF15 | 4.08 | | KIAA1549L | 7.10 | | MYH10 | -2.08 | | PSAPL1 | -7.95 | | SLC35A1 | 2.16 | | VEGFA | 2.19 |
| AC245100.3 | 4.63 | | BAIAP2-DT | -2.32 | | DEFB1 | -2.62 | | GDPD1 | 2.56 | | KIF21B | 2.41 | | MYLK3 | -3.10 | | PSCA | 2.28 | | SLC48A1 | 2.33 | | VGF | 4.44 |
| AC245100.6 | 6.63 | | BCAR3 | 2.53 | | DENND2D | 2.97 | | GIPC3 | -2.81 | | KLHDC7B | 3.52 | | MYLK-AS1 | 3.02 | | PSORS1C1 | 3.76 | | SLC4A3 | -2.59 | | VLDLR-AS1 | 2.72 |
| ACADSB | -2.24 | | BDKRB2 | -3.26 | | DEPDC1-AS1 | 7.01 | | GOLGA6L9 | 2.92 | | KLHL3 | 2.72 | | MYO7A | -2.16 | | PSPH | 2.16 | | SLC5A5 | -2.56 | | ZCCHC24 | -2.00 |
| ACAP1 | 2.08 | | BEST1 | 4.00 | | DES | -3.86 | | GOLGA8A | 2.08 | | KLHL31 | 3.39 | | NCCRP1 | -2.88 | | PXYLP1 | -2.24 | | SLC6A12 | -2.42 | | ZG16 | -2.16 |
| ADGRL2 | -2.43 | | BIRC3 | 2.41 | | DHRS2 | 9.16 | | GPLD1 | -3.91 | | KREMEN1 | -2.85 | | NDUFA4L2 | -3.67 | | PYGM | -3.96 | | SLC7A11 | 2.88 | | ZNF529-AS1 | 2.24 |
| ADIRF-AS1 | 2.07 | | BOLA2B | 2.97 | | DMGDH | 6.83 | | GPR1 | 3.30 | | KRT15 | -3.72 | | NEAT1 | 2.56 | | RAB7B | 2.55 | | SLC9A3-AS1 | 2.38 | | ZNF585A | 3.14 |
| ADM2 | 4.08 | | BTN3A3 | -2.11 | | DNAH3 | 7.62 | | GPR3 | 2.23 | | KRT34 | 3.34 | | NECAB2 | 4.13 | | RAPGEF3 | 3.36 | | SLCO2A1 | 3.88 | | ZNF862 | 7.51 |
| AGAP7P | 4.32 | | C1QL4 | -3.53 | | DUSP5 | 2.37 | | GPT | -3.79 | | LARGE2 | -3.36 | | NECTIN4 | 7.25 | | RARRES3 | 2.49 | | SMG1P5 | 2.05 | | | |
| AGR2 | 3.85 | | C6orf132 | 3.27 | | DUSP6 | 8.56 | | GRK4 | 2.13 | | LARP6 | 2.18 | | NES | -4.38 | | RBP4 | 3.59 | | SNHG1 | 2.35 | | | |
| AJUBA | 2.17 | | CABP7 | -4.81 | | ECM1 | 2.96 | | GSTA4 | -3.30 | | LETM2 | 4.99 | | NFATC4 | -2.72 | | RBPJL | 6.63 | | SPRED3 | 3.45 | | | |
| AKNA | 2.89 | | CACNA1A | 4.13 | | ECM2 | 5.89 | | GYG2 | -3.15 | | LIF | 2.36 | | NKD1 | -3.13 | | RCAN3 | -2.15 | | SPRY4 | 4.37 | | | |
| AL049796.1 | 3.00 | | CACNB2 | -2.10 | | EGFL8 | 6.63 | | HAPLN3 | -2.48 | | LINC00239 | 2.88 | | NLRP1 | 3.92 | | RF02250 | 6.63 | | SPSB4 | -3.82 | | | |
| AL096828.3 | 4.00 | | CALY | -2.74 | | EID3 | 2.93 | | HBA2 | -3.29 | | LINC002481 | 3.00 | | NMRAL2P | 2.38 | | RGS9 | -6.56 | | SPTB | -2.37 | | | |
| AL109615.3 | 2.23 | | CASC8 | 2.48 | | EIF4HP2 | 6.93 | | HHAT | 8.59 | | LINC00578 | -6.56 | | NOTUM | -2.69 | | RGS9BP | -3.26 | | SRGAP3 | -2.84 | | | |
| AL160254.1 | 3.24 | | CBSL | 7.67 | | ELOVL2 | -2.77 | | HIST1H2BO | 2.08 | | LINC00662 | 2.47 | | NPAS2 | 2.16 | | RHEBL1 | 2.48 | | SRPX2 | 2.36 | | | |
-9
12

## Slide 4
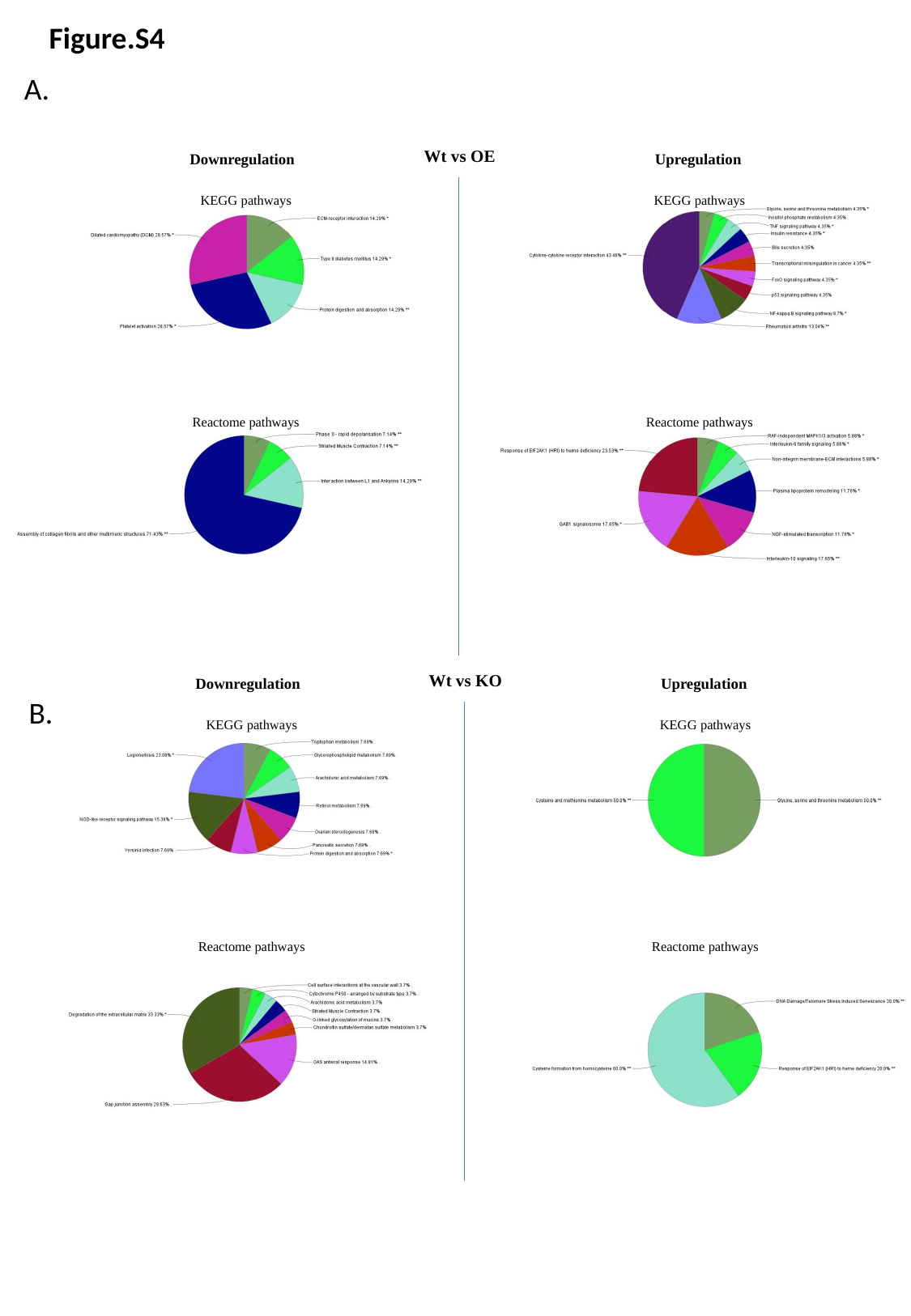

Figure.S4
A.
Wt vs OE
Downregulation
Upregulation
KEGG pathways
KEGG pathways
Reactome pathways
Reactome pathways
Wt vs KO
Downregulation
Upregulation
KEGG pathways
KEGG pathways
Reactome pathways
Reactome pathways
B.

## Slide 5
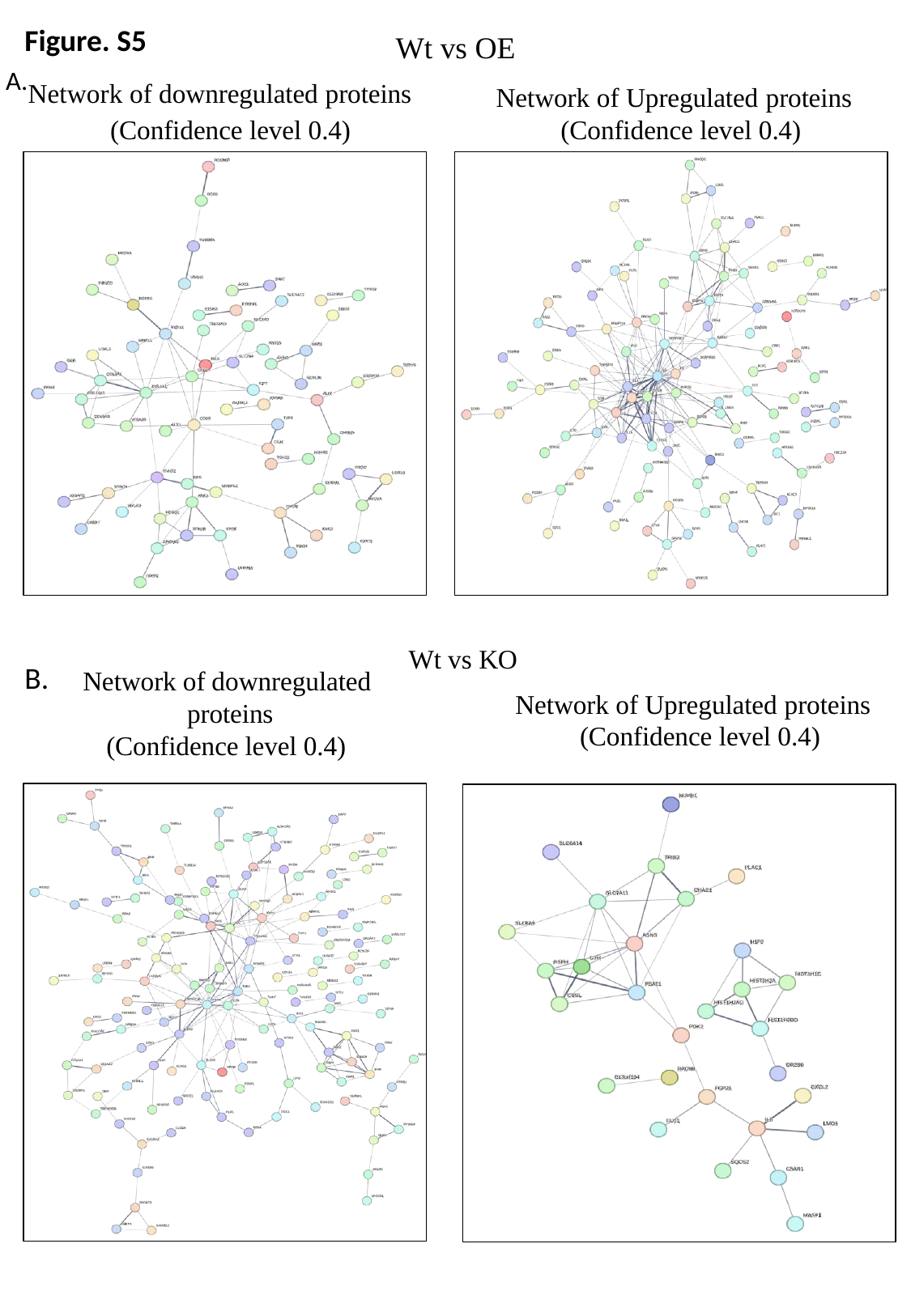

Figure. S5
Wt vs OE
A.Network of downregulated proteins (Confidence level 0.4)
Network of Upregulated proteins (Confidence level 0.4)
Wt vs KO
Network of Upregulated proteins (Confidence level 0.4)
B.
Network of downregulated proteins
(Confidence level 0.4)

## Slide 6
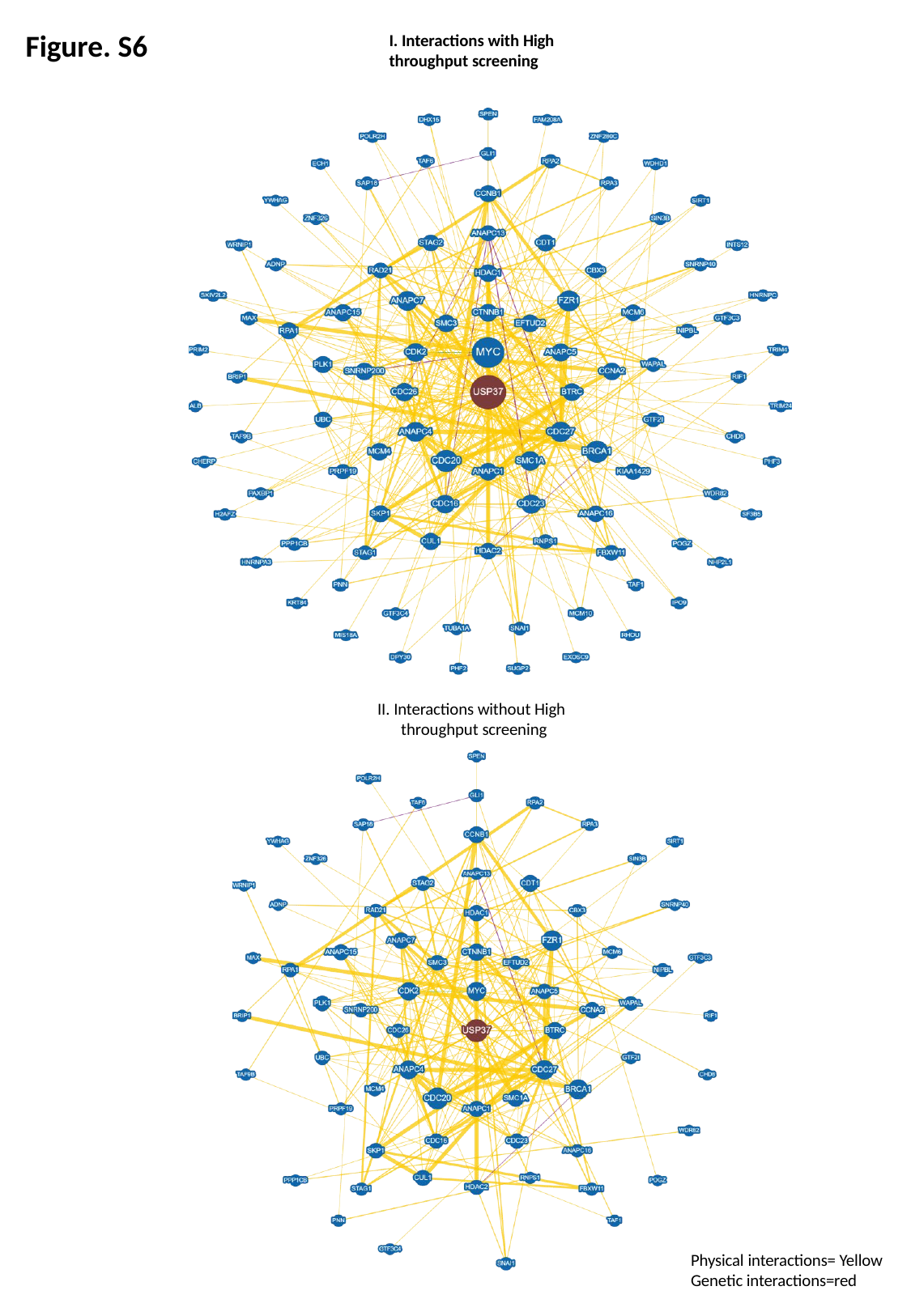

Figure. S6
I. Interactions with High throughput screening
II. Interactions without High throughput screening
Physical interactions= Yellow Genetic interactions=red

## Slide 7
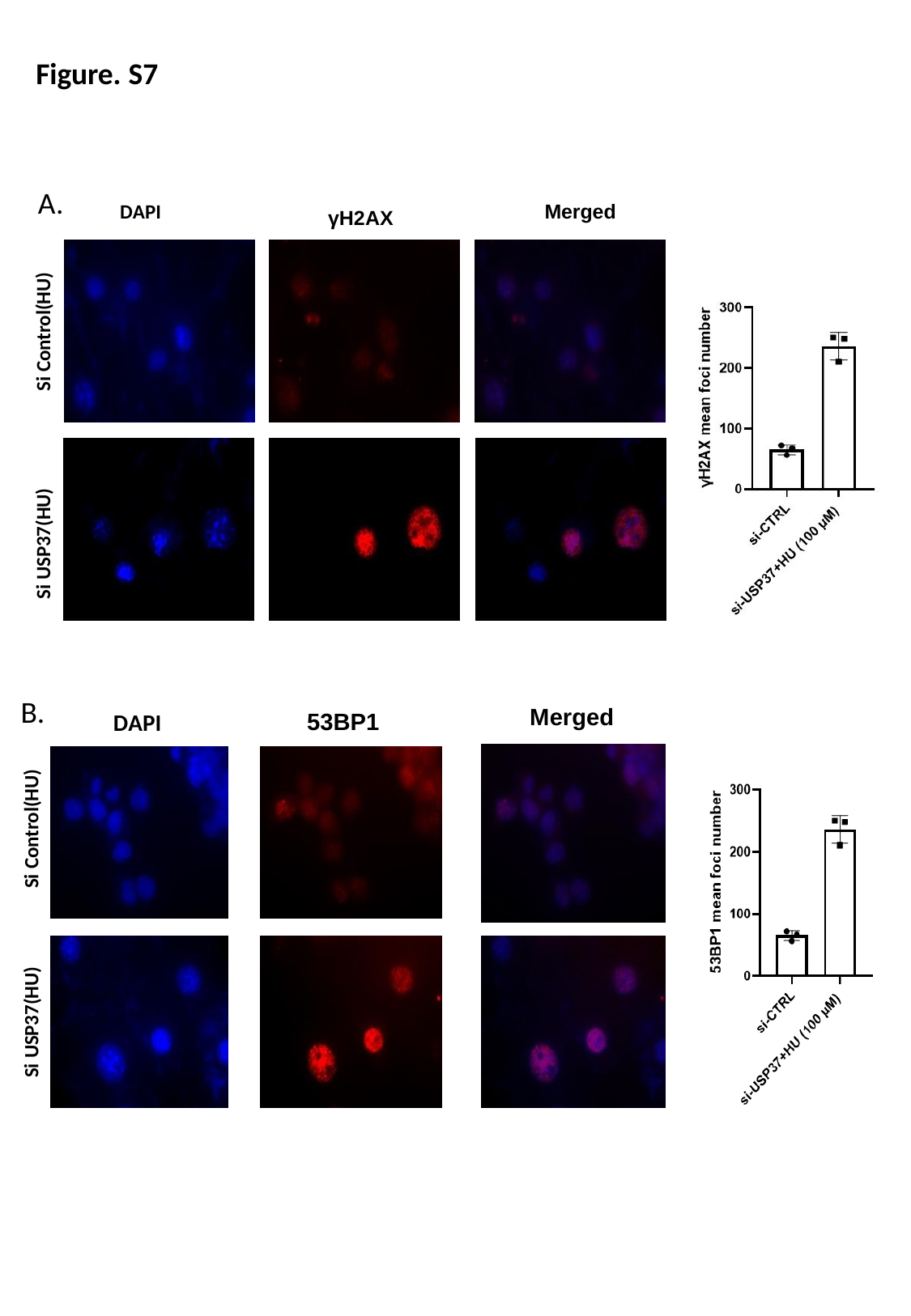

Figure. S7
A.
DAPI
Merged
γH2AX
Si Control(HU)
Si USP37(HU)
B.
Merged
53BP1
DAPI
Si Control(HU)
Si USP37(HU)

## Slide 8
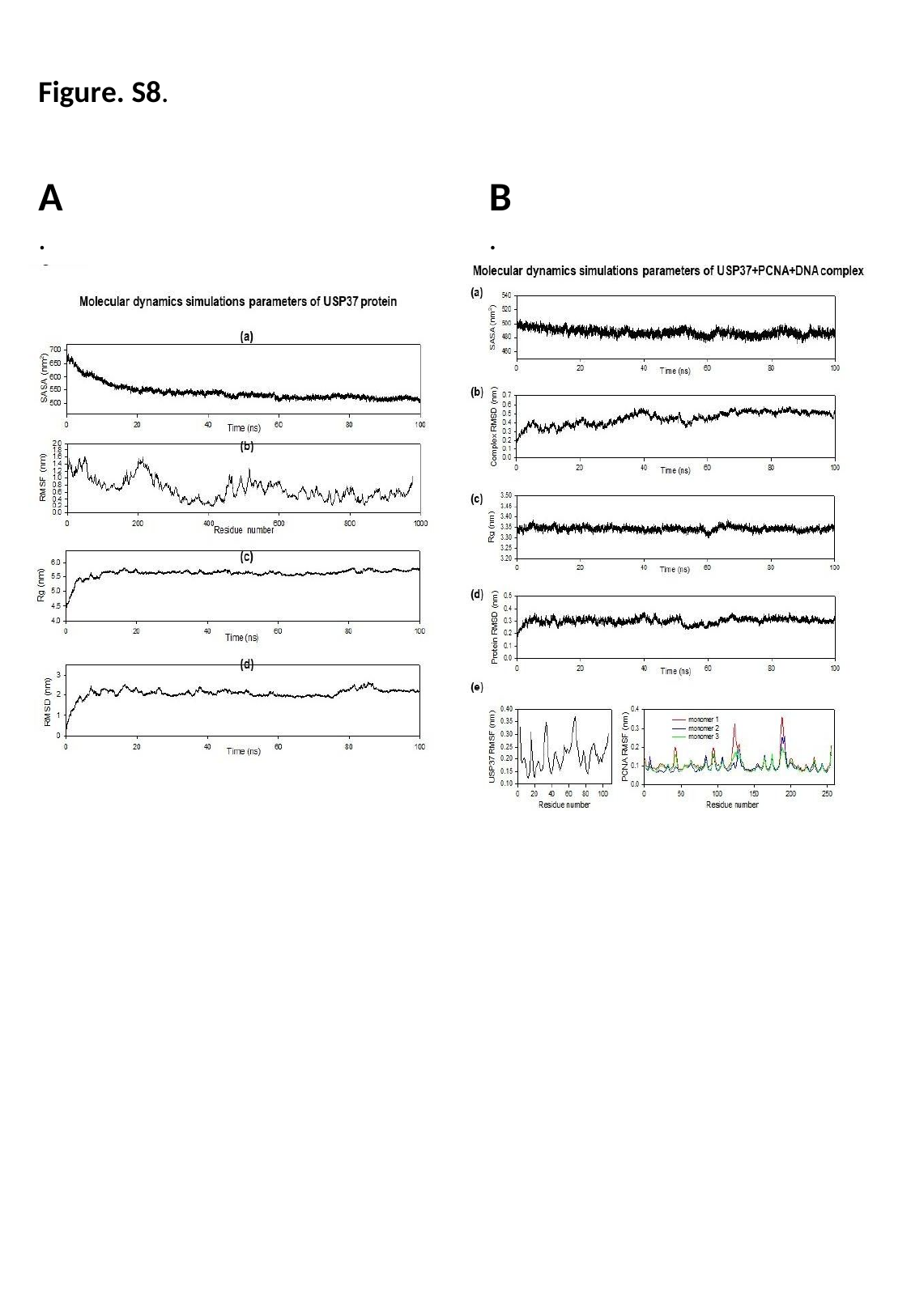

Figure. S8.
A
.
B
.
