# Supplementary material for: Ubiquitin specific peptidase 37 and PCNA interaction promotes osteosarcoma pathogenesis by modulating replication fork progression
Source: J Transl Med. 2023 Apr 28;21:286. doi: 10.1186/s12967-023-04126-2 (PMC10142227; doi:10.1186/s12967-023-04126-2)
Supplement: Supplementary file 2 — Additional file 2: Table S1. Reagent and Resources. [file 12967_2023_4126_MOESM2_ESM.docx]

**Table S1. Reagent and Resources**

| **Reagent or resource** | **Source** | **Identifier** |
| --- | --- | --- |
| **Antibodies** |  |  |
| USP37 Polyclonal Antibody (IHC) | Elabscience | Cat. E-AB-19606 |
| USP37 Polyclonal Antibody (WB) | Elabscience | E-AB-66492 |
| Anti-β-Actin Antibody | Santa Cruz Biotechnology | sc-47778 |
| Phospho-Histone H2A.X (Ser139) Antibody | Cell signalling technology | #2577 |
| Anti-53BP1 Antibody | Santa Cruz Biotechnology | sc-517281 |
| Anti-RPA 70 kDa subunit Antibody | Santa Cruz Biotechnology | (sc-28304) |
| PCNA (D3H8P) Rabbit mAb | Cell signalling technology | #13110 |
| Bax Antibody | Cell signalling technology | #2772 |
| Caspase-3 Antibody | Cell signalling technology | #9662 |
| Anti-mouse IgG, HRP-linked Antibody | Cell signalling technology | #7076 |
| Anti-rabbit IgG, HRP-linked Antibody | Cell signalling technology | #7074 |
| Donkey anti-Mouse IgG (H+L) Highly Cross-Adsorbed Secondary Antibody, Alexa Fluor 488 | ThermoFisher | Cat# A-21202 |
| 21206 Donkey anti-Rat IgG (H+L) Highly Cross-Adsorbed Secondary Antibody, Alexa Fluor 594 | ThermoFisher | Cat# A-21209 |
| U-2 OS [U2OS] Cell Line | Elabscience | Cat.No.:EP-CL-0236 |
| MG-63 Cell line | NCCS, Pune |  |
| MCF 10A Cell Line | Elabscience | Cat.No.:EP-CL-0525 |
| **Recombinant DNA** |  |  |
| pCS2-MYC-USP37 | Burrows, et al. 2012 |  |
| pCS2-TAP-USP37 C350A | Huang, et al. 2011 |  |
| **Critical Commercial Assays** |  |  |
| Click-IT EdU Imaging Kit | ThermoFisher | Cat# C10340, |
| Pierce BCA Protein Assay Kit | ThermoFisher | Cat# 23225 |
| NE-PER™ Nuclear and Cytoplasmic Extraction Reagents | Thermo Scientific | 78833 |
| UltraVisionQuanto Detection System HRP DAB | Thermo Scientific | TL-060-QHD |
